# Quantitative Analysis of OCT for Neovascular Age-Related Macular Degeneration Using Deep Learning

**DOI:** 10.1016/j.ophtha.2020.09.025

**Published:** 2021-05

**Authors:** Gabriella Moraes, Dun Jack Fu, Marc Wilson, Hagar Khalid, Siegfried K. Wagner, Edward Korot, Daniel Ferraz, Livia Faes, Christopher J. Kelly, Terry Spitz, Praveen J. Patel, Konstantinos Balaskas, Tiarnan D.L. Keenan, Pearse A. Keane, Reena Chopra

**Affiliations:** 1 NIHR Biomedical Research Centre for Ophthalmology, Moorfields Eye Hospital NHS Foundation Trust and UCL Institute of Ophthalmology, London, United Kingdom; 2 Google Health, London, United Kingdom; 3 Department of Ophthalmology, Federal University São Paulo, São Paulo, Brazil; 4 Division of Epidemiology and Clinical Applications, National Eye Institute, National Institutes of Health, Bethesda, Maryland

**Keywords:** age-related macular degeneration, neovascular, deep learning, OCT, automated, AI, artificial intelligence, AMD, age-related macular degeneration, CST, central subfield thickness, ELM, external limiting membrane, fvPED, fibrovascular pigment epithelium detachment, HRF, hyperreflective foci, IRF, intraretinal fluid, MNV, macular neovascularization, NSR, neurosensory retina, PED, pigment epithelium detachment, RPE, retinal pigment epithelium, sPED, serous pigment epithelial detachment, SD, standard deviation, SRF, subretinal fluid, SHRM, subretinal hyperreflective material, 3D, 3-dimensional, VA, visual acuity, VEGF, vascular endothelial growth factor

## Abstract

**Purpose:**

To apply a deep learning algorithm for automated, objective, and comprehensive quantification of OCT scans to a large real-world dataset of eyes with neovascular age-related macular degeneration (AMD) and make the raw segmentation output data openly available for further research.

**Design:**

Retrospective analysis of OCT images from the Moorfields Eye Hospital AMD Database.

**Participants:**

A total of 2473 first-treated eyes and 493 second-treated eyes that commenced therapy for neovascular AMD between June 2012 and June 2017.

**Methods:**

A deep learning algorithm was used to segment all baseline OCT scans. Volumes were calculated for segmented features such as neurosensory retina (NSR), drusen, intraretinal fluid (IRF), subretinal fluid (SRF), subretinal hyperreflective material (SHRM), retinal pigment epithelium (RPE), hyperreflective foci (HRF), fibrovascular pigment epithelium detachment (fvPED), and serous PED (sPED). Analyses included comparisons between first- and second-treated eyes by visual acuity (VA) and race/ethnicity and correlations between volumes.

**Main Outcome Measures:**

Volumes of segmented features (mm^3^) and central subfield thickness (CST) (μm).

**Results:**

In first-treated eyes, the majority had both IRF and SRF (54.7%). First-treated eyes had greater volumes for all segmented tissues, with the exception of drusen, which was greater in second-treated eyes. In first-treated eyes, older age was associated with lower volumes for RPE, SRF, NSR, and sPED; in second-treated eyes, older age was associated with lower volumes of NSR, RPE, sPED, fvPED, and SRF. Eyes from Black individuals had higher SRF, RPE, and serous PED volumes compared with other ethnic groups. Greater volumes of the majority of features were associated with worse VA.

**Conclusions:**

We report the results of large-scale automated quantification of a novel range of baseline features in neovascular AMD. Major differences between first- and second-treated eyes, with increasing age, and between ethnicities are highlighted. In the coming years, enhanced, automated OCT segmentation may assist personalization of real-world care and the detection of novel structure–function correlations. These data will be made publicly available for replication and future investigation by the AMD research community.

The advent of high-resolution in vivo OCT imaging has driven research to identify novel anatomic biomarkers in neovascular age-related macular degeneration (AMD).^1^, ^2^, ^3^ The upsurge in the number of patients requiring OCT scans for optimal macular disease management and increasing OCT scanning density have become a challenge.^3^ Automated tools that enable detailed analyses, including segmentation and quantification of features, may improve our understanding of neovascular AMD and could assist clinicians in making treatment decisions.

OCT-derived parameters such as central subfield thickness (CST) have been used to inform re-treatment decisions in clinical trials.^4^^,^^5^ Although basic measurements such as this can be automatically generated by OCT software algorithms at scale,^6^^,^^7^ multiple limitations have questioned their applicability to influence clinical decisions. These include susceptibility to segmentation errors,^8^ limited reproducibility between different OCT devices,^9^ and the lack of detailed information provided by CST measurement alone (which does not distinguish among neural tissue, retinal fluid, or retinal fluid in different compartments). Therefore, much attention has been given to identifying other OCT parameters for the optimal management of neovascular AMD.^2^ Post hoc analyses of clinical trials^10^, ^11^, ^12^, ^13^ and real-world studies^1^ have explored how certain baseline morphological parameters such as intraretinal fluid (IRF), subretinal fluid (SRF), subretinal hyperreflective material (SHRM), and pigment epithelium detachment (PED) may affect the structural and visual outcomes of patients beginning anti-vascular endothelial growth factor (VEGF) therapy.

Clinical trials such as Comparison of Age-Related Macular Degeneration Treatments Trials and HARBOR used macular fluid presence as a qualitative OCT parameter in their re-treatment protocols (in the pro re nata arms); this involved the manual detection of IRF or SRF from macular OCT scans.^14^^,^^15^ However, both qualitative and quantitative assessments demonstrate high rates of discrepancies between physicians and reading center experts, with disagreements on retinal fluid presence on OCT imaging.^15^ Recent advances in deep learning, a subfield of machine learning leveraging artificial neural networks, have stimulated an upsurge of automatic assessments of the different segmented features within an OCT volume, especially IRF, SRF, PED, and SHRM.^16^, ^17^, ^18^ Prior work using deep learning for fluid detection and segmentation has demonstrated highly accurate results and laid important groundwork for potential clinical and research applications.^19^, ^20^, ^21^, ^22^ Bogunovic et al^23^ conceived the RETOUCH challenge to spur the development of multi-class fluid segmentation models, recognizing that most research to date did not distinguish between the different fluid types within an OCT scan. This has been considered an important clinical limitation, because mounting evidence suggests that subtypes of macular fluid have distinct prognostic impacts on visual outcomes.^23^, ^24^, ^25^

In 2018, an artificial intelligence (AI)-derived system by De Fauw et al^26^ demonstrated applicability in diagnosing and triaging major retinal diseases, including neovascular AMD.^27^ In this report, we applied the system’s segmentation network component to the baseline OCT scans of eyes starting anti-VEGF therapy for neovascular AMD in the Moorfields Eye Hospital NHS Foundation Trust AMD Database.^28^^,^^29^ We use these segmentations to quantify a range of anatomic parameters and disease features and explore their potential significance. We also make these data publicly available for replication and future investigation by the AMD research community.

## Methods

### Dataset

The Moorfields AMD dataset for this study included all treatment-naive eyes that began anti-VEGF therapy for neovascular AMD between June 1, 2012, and June 30, 2017.^27^^,^^28^ Imaging data included macular OCT scans captured using 3DOCT-2000 devices (Topcon Corp, Tokyo, Japan), comprising 128 B-scans covering a volume of 6 × 6 × 2.3 mm. Patient demographics recorded in Moorfields’ electronic medical record, including age, self-reported gender identity, and race/ethnicity, along with associated clinical metadata including visual acuity (VA) in Early Treatment Diabetic Retinopathy Study (ETDRS) letters and whether an injection was administered, were also available for each visit. Whenever an OCT scan was not available on the exact day of the first injection for the first-treated eye, a scan from up to 14 days previously was used. Second-treated eyes that sequentially converted to neovascular AMD and started treatment in the time period of this study were also analyzed, with their baseline scan at their first injection visit used for analysis. Second-treated eyes were not required to have contributed to the first-treated eye cohort. All eyes were analyzed independently. If multiple scans were present on the same visit, the scan with the lowest volume of mirror and blink artefacts was selected for analysis. When neither of these artefacts existed, the scan with the lowest volume of padding artefact, indicating less manipulation performed by the OCT device software during postprocessing and therefore a cleaner image capture, was selected. Review and analysis of retrospective anonymized data were approved by the Moorfields Eye Hospital Institutional Review Board (ROAD17/031), and the research adhered to the tenets of the Declaration of Helsinki. All participants provided informed consent.

### Segmentation Network

All scans were input into the previously described 3-dimensional (3D) segmentation network.^26^ Briefly, the network automatically predicts segmented features present at each voxel based on a semantic segmentation architecture. Voxels can be summed and multiplied by the real-world voxel size to provide volumetric measurements of each feature in a 3D scan. For this study, the following segmented features were analyzed: neurosensory retina (NSR), retinal pigment epithelium (RPE), IRF, SRF, SHRM, hyperreflective foci (HRF), drusen, fibrovascular pigment epithelium detachment (fvPED), and serous PED (sPED). The NSR volume segmentation excluded the IRF, SRF, and SHRM components. As the segmentation network consists of an ensemble of 5 instances, the average voxel count between the instances was used.^26^ Each voxel equated to 2.60 × 11.72 × 47.24 μm in the A-scan, B-scan, and C-scan directions, respectively. These volumes were scaled to cubic millimeters for analysis. The CST measurements were defined as average thickness in the central 1-mm diameter circle of the ETDRS grid, measured in micrometers.^30^ The CST comprised all segmented features above the RPE to the inner boundary of the NSR, including SHRM, SRF, HRF, and IRF. For the binary classification of retinal fluid presence, the threshold at which fluid is definitely present from a clinical perspective was assessed. Two retinal specialists independently performed the binary classification task for IRF and SRF presence on a subset of 573 baseline scans, selected for gradability and to ensure coverage of the range of IRF/SRF segmented by the model. Because the segmentation model may contain some noise/error or subclinically relevant segmented volumes, this was important to determine the clinically relevant minimum voxel count (and the respective volumes) for both segmented features. The graders agreed on 524 of 573 scans (91.4%) for SRF presence and 487 of 573 scans (85.0%) for IRF presence (Fig S1, available at www.aaojournal.org). Receiver operating characteristic curves were plotted for the diagnostic accuracy of the segmentation model, using only scans where the retinal specialists agreed. The IRF and SRF were defined as present at ≥ 453 voxels (0.0007 mm^3^) and ≥ 5199 voxels (0.0075 mm^3^), respectively, based on the operating point closest to the upper left corner (Fig S2, available at www.aaojournal.org). Of 524 scans for which the experts agreed on the presence or absence of SRF, the model also agreed in 90.3% of scans. Of 487 scans for which the experts agreed on presence or absence of IRF, the model also agreed in 72.7% of scans (Fig S3, available at www.aaojournal.org).

### Statistical Analysis

The mean, standard deviation (SD), median, and interquartile range were calculated for each segmented feature, separately for first-treated and second-treated eyes. Boxplots were used to visualize the distribution of feature volumes between subgroups of eyes, according to age, race/ethnicity, VA, and first-treated versus second-treated eyes. These were displayed on a logarithmic scale to visualize a range in volume that spans several orders of magnitude between the segmented features. For the primary analyses, the relationships between first- and second-treated eyes, VA and feature volume, and age and feature volume were assessed. The distributions of the segmented features were non-normal, as assessed using the Shapiro–Wilk test. Nonparametric tests were used for statistical analysis. The Mann–Whitney *U* test was used to compare observed volumes between first-treated and second-treated eyes. Univariable regression and Spearman’s rank correlation were used to examine the associations between segmented features and age and VA, respectively. Statistical significance was set at *P* ≤ 0.05, with Bonferroni correction applied to the statistical tests in the regression and correlation analyses. The following analyses were considered exploratory. Stepwise multivariable linear regression was used to determine whether VA could be predicted using segmented features and demographic data: categoric variables were dummy coded, and backward elimination of features was used to determine significant variables with *P* ≤ 0.05. Kruskal–Wallis and post hoc Dunn’s tests were used for comparisons between ethnicities grouped into White, Asian, Black, and “Other or Unknown.” Spearman’s rank correlation coefficient was used to assess the relationships between paired feature volumes. All analysis was performed using Python 3.6. De-identified data for this study will be publicly available from the Dryad Digital Repository (https://doi.org/10.5061/dryad.2rbnzs7m4). The Moorfields Eye Hospital NHS Foundation Trust also intends to make the raw data shared with DeepMind openly available to researchers as part of the Ryan Initiative for Macular Research.^27^

## Results

**Figure 1:**
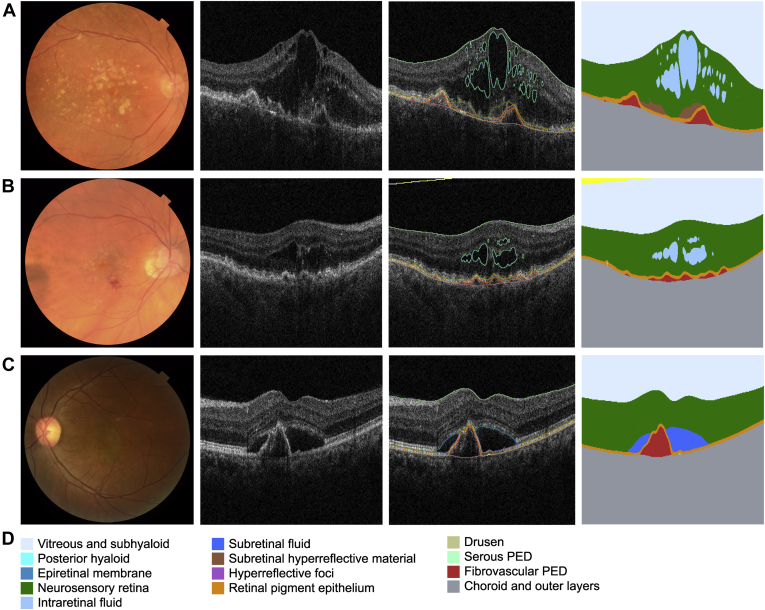
Fundus photograph, OCT scan, and corresponding segmentation map for 3 examples. **A,** Macular neovascularization (MNV) in a typical case of neovascular age-related macular degeneration (AMD): an 81-year-old White woman presenting with visual acuity (VA) of 63 Early Treatment Diabetic Retinopathy Study (ETDRS) letters. **B,** Type 3 MNV example: an 83-year-old woman of other/unknown ethnicity/race presenting VA of 70 ETDRS letters and OCT presenting intraretinal fluid (IRF) only. **C,** Polyp-like example in young patient: a 58-year-old Asian woman presenting VA of 59 ETDRS letters and OCT showing subretinal fluid (SRF) and suspicious polyp-like lesion. **D,** Color key for 13 anatomic features segmented by the segmentation network. PED = pigment epithelium detachment.

**Figure 2:**
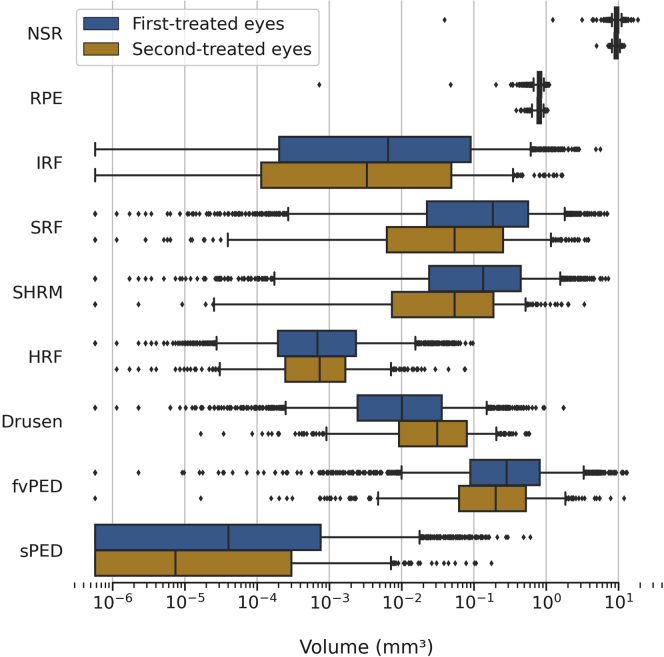
Distribution of segmented features volumes stratified by first- and second-treated eyes. The boxes show the median and interquartile range. Whiskers extend to the 5^th^ and 95^th^ percentiles, and beyond this outliers are shown individually. The volume (mm^3^) is distributed across a logarithmic scale; log(zero) is undefined, so zero values were set to the smallest positive value (5.8e-7). fvPED = fibrovascular pigment epithelium detachment; HRF = hyperreflective foci; IRF = intraretinal fluid; NSR = neurosensory retina; RPE = retinal pigment epithelium; SHRM = subretinal hyperreflective material; SRF = subretinal fluid; sPED = serous pigment epithelium detachment.

A total of 2966 baseline OCT scans from 2966 eyes of 2580 patients were evaluated. Of these images, 2473 (83.4%) were first-treated eyes and 493 (19.1%) were second-treated eyes. A total of 387 individuals contributed both a first-treated eye and second-treated eye to the analyses. The demographic characteristics of the patients are presented in Table 1. Example segmentations are shown in Figure 1. The volumes for each segmented feature are summarized in Table 2 and Figure 2. These baseline results are similar to those reported by other studies (Table S2, available at www.aaojournal.org).^3^^,^^31^, ^32^, ^33^, ^34^, ^35^, ^36^ The CST values are presented in Table S3 (www.aaojournal.org), alongside data collated from major clinical trials.^4^^,^^6^^,^^7^^,^^14^^,^^37^, ^38^, ^39^

**Table 1:** Demographics of Patients Included in Study

	First-Treated Eye	Second-Treated Eye
No. of eyes	2473	493
Gender		
Female (%)	1493 (60.4)	342 (69.4)
Male (%)	980 (39.6)	151 (30.6)
Race/Ethnicity		
White (%)	1319 (53.3)	290 (58.8)
Asian (%)	257 (10.4)	40 (8.1)
Black (%)	57 (2.3)	5 (1.0)
Other/Unknown (%)	840 (34.0)	158 (32.0)
Age (yrs)		
Mean (SD)	79.3 (8.6)	81.4 (7.9)
50–59 (%)	60 (2.4)	3 (0.6)
60–69 (%)	289 (11.7)	40 (8.1)
70–79 (%)	791 (32.0)	139 (28.2)
≥80 (%)	1332 (53.9)	311 (63.1)
VA (ETDRS letters)		
Mean (SD)	54.0 (16.1)	62.5 (13.2)
0–35 (%)	385 (15.6)	27 (5.5)
36–52 (%)	506 (20.5)	64 (13.0)
5369 (%)	885 (35.8)	202 (41.0)
≥70 (%)	471 (19.0)	194 (39.4)
Unknown VA (%)	226 (9.2)	6 (1.2)

SD = standard deviation; VA = visual acuity.

**Table 2:** Baseline Mean and Median Volumes of OCT Segmented Features in First- and Second-Treated Eyes

Segmented Feature	Mean (SD) at First Injection		Median (IQR) at First Injection		Mann–Whitney *U* Test *P* Value
	First-Treated Eye	Second-Treated Eye	First-Treated Eye	Second-Treated Eye	
NSR volume (mm^3^)	**9.485 (1.013)**	9.269 (0.775)	**9.445 (8.905–9.983)**	9.306 (8.790–9.767)	<0.001
RPE volume (mm^3^)	**0.806 (0.094)**	0.794 (0.088)	**0.808 (0.763–0.857)**	0.800 (0.755–0.845)	0.002
IRF volume (mm^3^)	**0.118 (0.309)**	0.073 (0.196)	**0.007 (0.000–0.090)**	0.003 (0.000–0.049)	<0.001
SRF volume (mm^3^)	**0.455 (0.733)**	0.258 (0.532)	**0.183 (0.022–0.562)**	0.054 (0.006–0.252)	<0.001
SHRM volume (mm^3^)	**0.380 (0.661)**	0.148 (0.283)	**0.135 (0.024–0.445)**	0.054 (0.007–0.186)	<0.001
HRF volume (mm^3^)	**0.003 (0.008)**	0.002 (0.006)	**0.001 (0.000–0.002)**	0.001 (0.000–0.002)	0.318
Drusen volume (mm^3^)	0.036 (0.085)	**0.060 (0.080)**	0.010 (0.002–0.036)	**0.031 (0.009–0.080)**	<0.001
fvPED volume (mm^3^)	**0.765 (1.305)**	0.491 (0.935)	**0.283 (0.089–0.815)**	0.200 (0.062–0.523)	<0.001
sPED volume (mm^3^)	**0.004 (0.023)**	0.002 (0.012)	**0.000 (0.000–0.001)**	0.000 (0.000–0.000)	<0.001
CST (μm)	**347.1 (114.3)**	306.1 (85.1)	**325.8 (266.6–405.0)**	295.0 (253.9–340.3)	<0.001

CST = central subfield thickness; fvPED = fibrovascular PED; HRF = hyperreflective foci; IRF = intraretinal fluid; IQR = interquartile range; NSR = neurosensory retina; PED = pigment epithelium detachment; RPE = retinal pigment epithelium; SD = standard deviation; SHRM = subretinal hyperreflective material; sPED = serous PED; SRF = subretinal fluid.

Segmented voxels were converted into mm^3^. *P* values were considered significant at ≤ 0.05.

Boldface values indicate greater mean volumes comparing first eyes versus second eyes. Only drusen had higher volume in the second eye.

### First-Treated versus Second-Treated Eyes

Significant differences in baseline volumes between first-treated and second-treated eyes were observed for every segmented feature analyzed except HRF (Table 2). With the exception of drusen, first-treated eyes had greater volumes of all features (Fig 2). The mean CST for first-treated and second-treated eyes was 347.1 μm (SD, 114.3) and 306.1 μm (SD, 85.1), respectively, and was significantly different (*P <* 0.001). Volumes in individuals with both first- and second-treated eyes (n = 387) are presented in Table S1 (available at www.aaojournal.org).

### Correlations between Segmented Features

**Figure 3:**
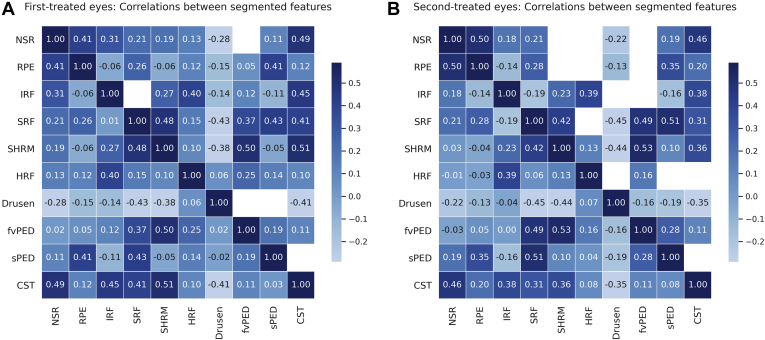
Spearman’s correlation between segmented feature volumes and central subfield thickness (CST) for **(A)** first- and **(B)** second-treated eyes. Tiles display the coefficient *r*_*s*_. The upper right half blanks out tiles that have a *P* > 0.05; values are symmetrical otherwise. fvPED = fibrovascular pigment epithelium detachment; HRF = hyperreflective foci; IRF = intraretinal fluid; NSR = neurosensory retina; RPE = retinal pigment epithelium; SHRM = subretinal hyperreflective material; sPED = serous pigment epithelium detachment; SRF = subretinal fluid.

The coefficients of Spearman’s correlation analyses between paired segmented features are presented as matrices in Figure 3. The FvPED and SHRM volumes were moderately and positively correlated with each other, and with SRF volume, in both first- and second-treated eyes but poorly correlated with IRF volume in first-treated eyes. The RPE volume showed a moderate positive correlation with NSR and sPED volumes in both sets of eyes. Hyperreflective foci showed the strongest volumetric correlation with IRF and vice versa.

### Volumes and Visual Acuity

**Figure 4:**
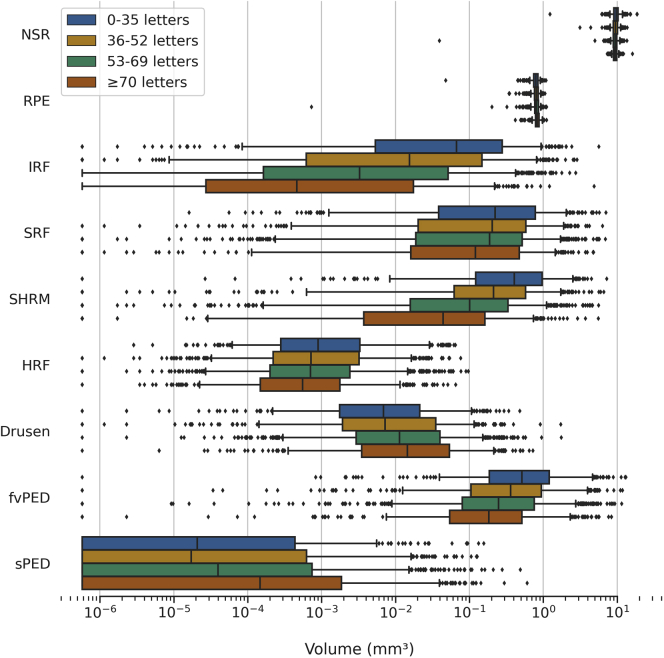
Distribution of first-treated eye segmented feature volumes stratified by baseline visual acuity (VA) subgroups. The VA is stratified into Early Treatment Diabetic Retinopathy Study (ETDRS) letters of 0–35, 36–52, 53–69, and 70 or greater (Table S4, available at www.aaojournal.org). The boxes show the median and interquartile range. Whiskers extend to the 5^th^ and 95^th^ percentiles, and beyond this outliers are shown individually. The volume is distributed across a logarithmic scale; log(zero) is undefined, so zero values were set to the smallest positive value (5.8e-7). fvPED = fibrovascular pigment epithelium detachment; HRF = hyperreflective foci; IRF = intraretinal fluid; NSR = neurosensory retina; RPE = retinal pigment epithelium; SHRM = subretinal hyperreflective material; sPED = serous pigment epithelium detachment; SRF = subretinal fluid.

The distributions of segmented features volumes in first-treated eyes, stratified by VA subgroups, are shown in Figure 4. The mean volumes of first-treated eyes, stratified by VA, age, and race/ethnicity subgroups, are summarized in Table S4 (available at www.aaojournal.org) and discussed in detail in the following sections.

In first-treated eyes (Table 3), all segmented features had weak negative volumetric correlations with VA (each *P <* 0.001), with the exception of sPED, RPE, and drusen, which presented weak positive correlations. Univariable linear regression analysis showed CST had the greatest association with VA (*R*^*2*^ = 0.107, *P <* 0.001) of all features considered (Fig S4, available at www.aaojournal.org). The strongest volumetric correlation was observed between SHRM and VA for both first- and second-treated eyes (*r*_*s*_ = −0.380, *P <* 0.001 and *r*_*s*_ = −0.293, *P <* 0.001, respectively). Likewise, univariable linear regression showed SHRM had the greatest association with VA in second-treated eyes (*R*^*2*^ = 0.122, *P <* 0.001) (Fig S5, available at www.aaojournal.org). Apart from NSR, sPED, RPE, and drusen, which had positive correlations with VA, all other volumes had weakly negative correlations with VA in second-treated eyes (Table 4). Drusen and NSR did not remain significant post-Bonferroni correction. The SRF and HRF were not found to be significantly correlated with VA in second-treated eyes.

**Table 3:** Univariable Linear Regression and Spearman’s Rank Correlation Coefficient Assessing the Relationship between Volumes and Visual Acuity, and Age and Volumes, in First-Treated Eyes

Volumes (X), Visual Acuity (Y)	Linear Regression (Ordinary Least Squares)				Spearman’s Rank	
	R^2^	Coefficient	Intercept	P Value	r_s_	*P* Value
CST†	0.107	−0.045	69.855	**<0.001**∗	−0.306	**<0.001**∗
SHRM	0.082	−7.013	56.616	**<0.001**∗	−0.380	**<0.001**∗
IRF	0.054	−11.939	55.410	**<0.001**∗	−0.347	**<0.001**∗
RPE	0.027	30.273	29.548	**<0.001**∗	0.169	**<0.001**∗
fvPED	0.022	−1.824	55.389	**<0.001**∗	−0.210	**<0.001**∗
NSR	0.015	−1.957	72.530	**<0.001**∗	−0.088	**<0.001**∗
SRF	0.008	−1.947	54.866	**<0.001**∗	−0.090	**<0.001**∗
Drusen	0.008	16.637	53.370	**<0.001**∗	0.144	**<0.001**∗
HRF	0.005	−141.423	54.456	**<0.001**∗	−0.092	**<0.001**∗
sPED	0.005	50.395	53.753	**<0.001**∗	0.134	**<0.001**∗

Age (X), Volumes (Y)	Linear Regression (Ordinary Least Squares)				Spearman’s Rank	
	R^2^	Coefficient	Intercept	P Value	r_s_	*P* Value
RPE	0.061	−0.003	1.005	**<0.001**∗	−0.257	**<0.001**∗
sPED	0.013	0.000	0.028	**<0.001**∗	−0.218	**<0.001**∗
NSR	0.010	−0.012	10.415	**<0.001**∗	−0.114	**<0.001**∗
SRF	0.006	−0.007	0.988	**<0.001**∗	−0.140	**<0.001**∗
IRF	0.004	0.002	−0.069	**0.001**∗	0.171	**<0.001**∗
HRF	0.001	0.000	0.001	0.064	0.056	**0.005**
fvPED	0.001	−0.004	1.091	0.178	0.020	0.323
SHRM	0.001	0.002	0.223	0.200	0.026	0.203
CST†	0.000	−0.234	372.987	0.391	−0.011	0.578
Drusen	0.000	0.000	0.028	0.603	0.117	**<0.001**∗

CST = central subfield thickness; fvPED = fibrovascular pigment epithelium detachment; HRF = hyperreflective foci; IRF = intraretinal fluid; NSR = neurosensory retina; RPE = retinal pigment epithelium; SHRM = subretinal hyperreflective material; sPED = serous pigment epithelium detachment; SRF = subretinal fluid.

*P* values are given before Bonferroni correction. Bold values were significant at *P* ≤ 0.05. Asterisked (∗) *P* values remain significant at *P* ≤ 0.005 after Bonferroni correction.

† CST measures thickness and not volume.

**Table 4:** Univariable Linear Regression and Spearman’s Rank Correlation Coefficient Assessing the Relationship between Volumes and Visual Acuity, and Age and Volumes, in Second-Treated Eyes

Volumes (X), Visual Acuity (y)	Linear Regression (Ordinary Least Squares)				Spearman’s Rank	
	R^2^	Coefficient	Intercept	P Value	r_s_	*P* Value
SHRM	0.122	−16.23	64.976	**<0.001**∗	−0.293	**<0.001**∗
RPE	0.067	39.859	30.888	**<0.001**∗	0.239	**<0.001**∗
CST†	0.024	−0.02	69.932	**<0.001**∗	−0.152	**0.001**∗
IRF	0.023	−10.17	63.315	**<0.001**∗	−0.224	**<0.001**∗
fvPED	0.020	−2.00	63.549	**0.002**∗	−0.142	**0.002**∗
NSR	0.016	2.20	42.140	**0.006**	0.114	**0.012**
SRF	0.014	−2.89	63.318	**0.010**	−0.020	0.659
Drusen	0.010	16.72	61.573	**0.028**	0.118	**0.009**
HRF	0.004	−139.85	62.853	0.170	−0.089	−0.089
sPED	0.002	47.21	62.466	0.354	0.136	**0.003**∗

Age (X), Volumes (y)	Linear Regression (Ordinary Least Squares)				Spearman’s Rank	
	R^2^	Coefficient	Intercept	P Value	r_s_	P Value
RPE	0.031	−0.002	0.952	**<0.001**∗	−0.245	**<0.001**∗
NSR	0.031	−0.017	10.670	**<0.001**∗	−0.171	**<0.001**∗
IRF	0.014	0.003	−0.167	**0.008**	0.190	**<0.001**∗
sPED	0.013	0.000	0.016	**0.011**	−0.209	**<0.001**∗
SRF	0.012	−0.007	0.849	**0.016**	−0.174	**<0.001**∗
fvPED	0.009	−0.011	1.398	**0.036**	−0.111	**0.014**
CST†	0.002	−0.445	348.885	0.367	−0.031	0.493
HRF	0.001	0.000	0.004	0.483	0.103	**0.022**
SHRM	0.000	0.000	0.187	0.767	−0.034	0.453
Drusen	0.000	0.000	0.059	0.978	0.068	0.132

CST = central subfield thickness; fvPED = fibrovascular pigment epithelium detachment; HRF = hyperreflective foci; IRF = intraretinal fluid; NSR = neurosensory retina; RPE = retinal pigment epithelium; SHRM = subretinal hyperreflective material; sPED = serous pigment epithelium detachment; SRF = subretinal fluid.

*P* values are given before Bonferroni correction.

∗ Remains significant at *P* ≤ 0.005 after Bonferroni correction (boldface).

† Central subfield thickness measures thickness and not volume.

Multivariable linear regression analysis, with VA as the dependent variable, yielded a model with adjusted *R*^*2*^ = 0.209 for first-treated eyes. All feature volumes, CST, age, gender, and race/ethnicity were used in the initial model. Stepwise regression eliminated NSR and sPED, and all 14 remaining variables were significant (*P <* 0.05) (Table S5, available at www.aaojournal.org).

### Volumes and Age

**Figure 5:**
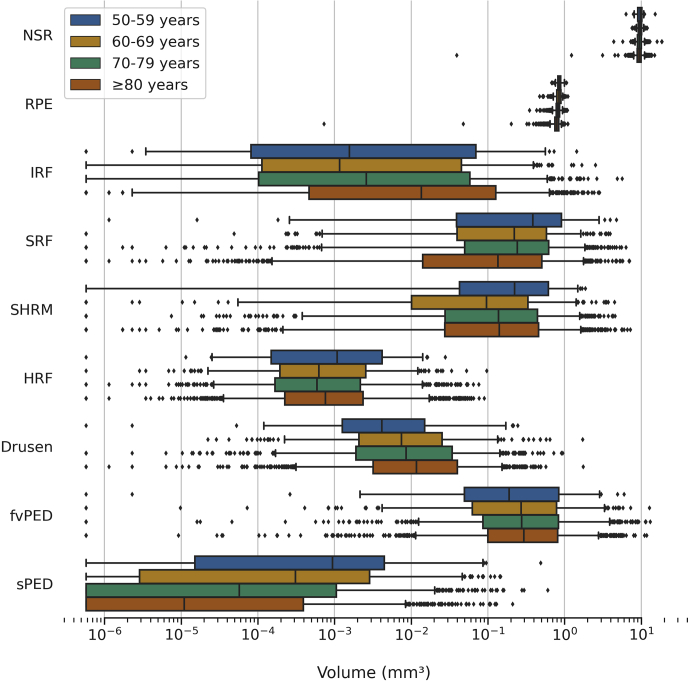
Distribution of segmented feature volumes in the first-treated eye at baseline stratified by age groups 50–59, 60–69, 70–79, and ≥80 years (Table S4, available at www.aaojournal.org) across a logarithmic scale; log(zero) is undefined, so zero values were set to the smallest positive value (5.8e-7). The boxes show the median and interquartile range. Whiskers extend to the 5^th^ and 95^th^ percentiles, and beyond this outliers are shown individually. fvPED = fibrovascular pigment epithelium detachment; HRF = hyperreflective foci; IRF = intraretinal fluid; NSR = neurosensory retina; RPE = retinal pigment epithelium; SHRM = subretinal hyperreflective material; sPED = serous pigment epithelium detachment; SRF = subretinal fluid.

The distributions of segmented feature volumes in first-treated eyes, stratified by age groups, are shown in Figure 5. Mean volumes of first-treated eyes are summarized in Table S4 (available at www.aaojournal.org).

In first-treated eyes (Table 3), weak negative volumetric correlations between age and RPE (*r*_*s*_ = −0.257, *P <* 0.001), sPED (*r*_*s*_ = −0.218, *P <* 0.001), NSR (*r*_*s*_ = −0.114, *P <* 0.001), and SRF (*r*_*s*_ = −0.140, *P <* 0.001) were observed. The IRF and drusen were significantly positively correlated with age (*r*_*s*_ = 0.171 and *r*_*s*_ = 0.117, respectively). Univariable linear regression analysis showed RPE had the greatest association with age (*R*^*2*^ = 0.061, *P <* 0.001) of all features considered. In second-treated eyes (Table 4), parameters that had weak negative correlations with age were NSR (*r*_*s*_ = −0.171, *P <* 0.001), RPE (*r*_*s*_ = −0.245, *P <* 0.001), sPED (*r*_*s*_ = −0.209, *P <* 0.001), fvPED (*r*_*s*_ = −0.111, *P* = 0.014), and SRF (*r*_*s*_ = −0.174, *P <* 0.001). Similar to first-treated eyes, IRF in second-treated eyes also had a significantly positive correlation with age (*r*_*s*_ = 0.190, *P <* 0.001). Univariable linear regression analysis showed RPE and NSR had the greatest association with age (*R*^*2*^ = 0.031, *P <* 0.001 for both segmented features) of all features considered.

### Volumes and Race/Ethnicity

**Figure 6:**
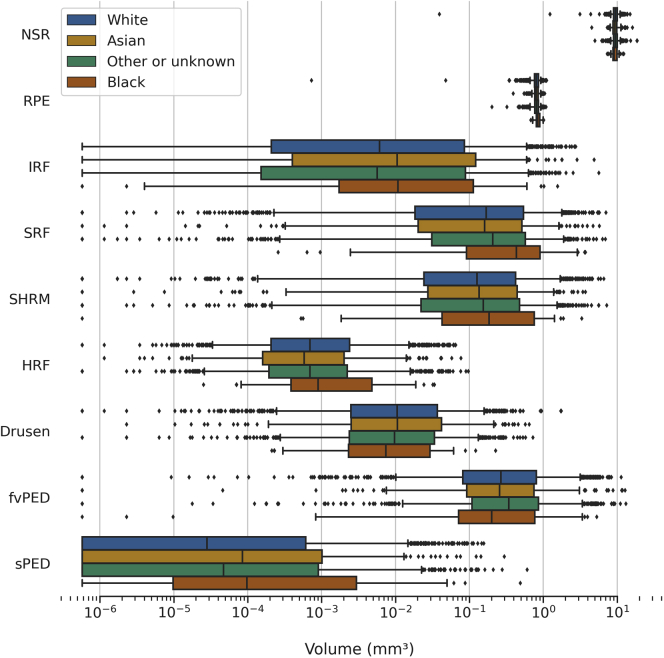
Distribution of segmented feature volumes in the first-treated eyes at baseline stratified by ethnicities: White, Asian, other or unknown, and Black (Table S4, available at www.aaojournal.org). The boxes show the median and interquartile range. Whiskers extend to the 5^th^ and 95^th^ percentiles, and beyond this outliers are shown individually. The volume is distributed across a logarithmic scale; log(zero) is undefined, so zero values were set to the smallest positive value (5.8e-7). fvPED = fibrovascular pigment epithelium detachment; HRF = hyperreflective foci; IRF = intraretinal fluid; NSR = neurosensory retina; RPE = retinal pigment epithelium; SHRM = subretinal hyperreflective material; sPED = serous pigment epithelium detachment; SRF = subretinal fluid.

The distributions of segmented feature volumes in the first-treated eyes, stratified by race/ethnicity, are shown in Figure 6. Mean volumes of first-treated eyes are summarized in Table S4 (available at www.aaojournal.org). Significant differences in volumes for RPE, SRF, fvPED, and sPED were found between the different ethnic groups. Eyes from Black patients had significantly higher volumes of SRF (*P <* 0.05) and RPE (*P <* 0.05) than all other groups and greater sPED volumes when compared with White and other/unknown ethnicities (*P <* 0.05). For fvPED, only volumes in White patients versus other/unknown patients were significant in post hoc tests (*P <* 0.05).

### Presence of IRF and SRF at Baseline

The results of the proportion of eyes with IRF or SRF present (considered qualitatively, as present or absent) at baseline are displayed in Table 5. These results were compared with those from major clinical trials (Table S6, available at www.aaojournal.org).^4^^,^^11^^,^^33^^,^^39^ Intraretinal fluid was present in 66.8% and 60.2% of first- and second-treated eyes, respectively, whereas SRF was present in 82.7% and 72.6%, respectively. In first-treated eyes, the majority of eyes had both IRF and SRF (54.7%). For both sets of eyes, a greater number of eyes had SRF alone (28.0% in first-treated eyes and 33.7% in second-treated eyes) versus IRF alone (12.2% in first-treated eyes and 21.3% in second-treated eyes).

**Table 5:** Relative Presence of IRF and SRF at Baseline

Parameter	First-Treated Eye (Total n = 2473 Eyes)	Second-Treated Eye (Total n = 493 Eyes)
IRF [n, (%)]	1653 (66.8)	297 (60.2)
SRF [n, (%)]	2045 (82.7)	358 (72.6)
IRF only without SRF [n, (%)]	301 (12.2)	105 (21.3)
SRF only without IRF [n, (%)]	693 (28.0)	166 (33.7)
IRF and SRF [n, (%)]	1352 (54.7)	192 (38.9)
Neither IRF nor SRF [n, (%)]	127 (5.1)	30 (6.1)

IRF = intraretinal fluid; SRF = subretinal fluid.

Intraretinal fluid and SRF are defined as present at ≥ 453 voxels (0.0007 mm^3^) and ≥ 5199 (0.0075 mm^3^) voxels, respectively.

## Discussion

The accumulation of vast quantities of imaging data has become both a major challenge and an exciting opportunity for ophthalmology in the 21st century. At Moorfields Eye Hospital alone, there has been a substantial 14-fold increase in the number of OCT scans captured per year since 2008, from 23 582 scans to 339 639 in 2016.^40^ Artificial intelligence, through the use of machine learning methods, has the potential to revolutionize retinal diagnostics with techniques that may help optimize disease management and offer the possibility of more personalized medicine.^24^^,^^41^^,^^42^ In this study, we applied a deep learning–based segmentation algorithm to OCT scans from the Moorfields AMD Database to automatically identify and quantify multiple OCT features.

The IRF, SRF, PED, and SHRM are important indicators of disease activity in macular neovascularization (MNV). With the use of our clinical threshold of fluid presence, the majority of eyes had both IRF and SRF present at the time of diagnosis in first-treated eyes. The fluid volumes demonstrated a wide distribution, particularly for IRF, and were likely influenced by different lesion types,^2^^,^^43^ variability of lesion size and activity, differences in speed of patient presentation, and other physiologic factors such as VEGF levels, RPE pump function, and integrity of the blood-retinal barriers. Few eyes had IRF alone, likely arising from type 3 MNV or from VEGF-induced leakage from intraretinal vessels (Fig 1B).^43^ The IRF volume had a weak but significant positive correlation with age. Older patients may have a higher threshold for noticing and acting on visual symptoms and may either struggle or do not have the adequate support to access eye care, leading to delayed hospital visits and later presentation of the disease.^44^^,^^45^ In addition, these patients may be more likely to have more IRF than younger patients due to lower external limiting membrane (ELM) integrity or the presence of type 3 MNV. The negative prognostic impact that both increased IRF and older age independently have on visual outcomes has been well documented.^10^^,^^11^^,^^28^^,^^46^

Approximately one-fourth of first-treated eyes had SRF alone, likely representing a mixture of type 1 MNV (where SRF is thought to be the first exudative sign) and type 2 MNV (particularly when the ELM is intact).^2^^,^^43^^,^^47^ In contrast to IRF, SRF volume had a significant negative correlation with age. The younger population in our study tended to demonstrate greater volumes of SRF and sPED. Black individuals had a significantly higher volume of SRF and RPE than all other ethnic groups and more sPED than all other groups except Asian individuals. Younger patients may be more likely to present sooner, to have an intact ELM, and to have type 1 or 2 MNV rather than type 3. Some of these cases may even represent polypoidal choroidal vasculopathy, which characteristically presents with SRF and sPED and is more common in younger and Black and Asian populations (Fig 1C).^48^ This is closely linked to our findings on correlation between segmented features, where sPED volume showed moderate correlations with both SRF and RPE volumes.

Visual acuity was more strongly associated with IRF than SRF, consistent with previous studies.^1^^,^^3^^,^^10^^,^^31^ Greater IRF volume at baseline has been shown to be more detrimental to VA than SRF.^1^^,^^11^^,^^17^^,^^49^, ^50^, ^51^ The importance of differentiating among fluid types has been considered in clinical trials. In the FLUID study, tolerating some SRF, but not IRF, resulted in VA outcomes that were noninferior and involved fewer injections.^25^ In the Comparison of Age-Related Macular Degeneration Treatments Trials, IRF was associated with double the risk of geographic atrophy development.^52^ Consistent with other reports,^32^ there was a moderate negative correlation between VA and SHRM for both first- and second-treated eyes at baseline, supporting the idea that SHRM forms a mechanical barrier between the RPE and photoreceptors that disrupts the visual cycle.^13^

Our comparison between first- and second-treated eyes at their first injection visit revealed that second-treated eyes had significantly smaller volumes of IRF, SRF, SHRM, fvPED, and sPED compared with first-treated eyes, suggesting detection at an earlier stage of the disease. A later presentation in first-treated eyes may be associated with a more advanced stage of lesion maturity and higher degrees of fibrosis or atrophy. This is likely related to the close surveillance of second-treated eyes, whereas first-treated eyes are undergoing treatment; neovascular conversion in second-treated eyes might be detected at an earlier stage, even before the onset of visual symptoms.^29^^,^^53^ Furthermore, systemic absorption of anti-VEGF drugs has been suggested to decrease VEGF activity in second-treated eyes, possibly resulting in decreased exudation.^54^, ^55^ Drusen was the only segmented feature that presented greater volumes in second-treated eyes when compared with first-treated eyes (*P <* 0.05). This could be explained not only by earlier disease detection in second-treated eyes but also by the natural progression of dry AMD before conversion, which in both cases results in a greater drusen volume.

In both first- and second-treated eyes, fvPED volume correlated moderately with SRF volume and correlated poorly (first-treated eyes) or did not correlate (second-treated eyes) with IRF volume. This may relate to the pathophysiology of each fluid type, where SRF presumably arises directly from a vascularized PED, but IRF may come from a vascularized PED but may also arise from leakage from intraretinal vasculature or a type 3 MNV.^43^^,^^47^ Additionally, SHRM volume correlated moderately with SRF volume, which presumably relates to the broader definition of SHRM as the exudation of various materials such as serum, fibrin, and inflammatory cells into the subretinal space,^43^^,^^56^ and SRF being closely associated with some of these materials.

Hyperreflective foci showed the strongest volumetric correlation with IRF and vice versa. The origins of HRF in neovascular AMD are unclear, but one hypothesis is that they represent intraretinal hard exudates secondary to disruption of the blood–retinal barrier,^57^ which could explain their association with IRF. Hyperreflective foci have been shown to be a negative prognostic indicator, and their presence in various retinal layers at baseline has been associated with poor VA.^58^^,^^59^ Results from this study show that, although weak, HRF had a negative correlation with VA at baseline for both first-treated eyes and second-treated eyes.

The NSR volume had a moderate positive correlation with RPE volume in both first- and second-treated eyes. In cases in which macular atrophy accompanies neovascular AMD, lower volumes of both RPE and NSR might be observed.^43^ Although in first-treated eyes, NSR volume was negatively correlated with VA, it was positively correlated in second-treated eyes. This likely reflects the effect that several different layers may have on NSR thickening or thinning. On one hand, thickening from noncystic IRF leads to higher NSR volumes, whereas outer retinal atrophy leads to lower NSR volumes, both associated with worse VA.^33^^,^^34^ The RPE volume was also moderately correlated with SRF volume in both sets of eyes. It has been proposed that the presence of SRF due to an adjacent perfused neovascular net and functional choriocapillary layer promotes a favorable environment for a viable RPE.^2^ The RPE volumes were significantly positively correlated with VA in both first- and second-treated eyes, reflecting poorer vision in eyes with RPE loss, and thus atrophy.

Pigment epithelium detachment has increasingly been considered a relevant parameter for progressive neovascular activity. There is no consistent defining criteria for PED among studies, and most do not classify the PED by subtype.^60^ The AI system used in this study automatically subcategorized PED into fvPED and sPED. The disadvantage of including them within the same category has been discussed because of their different effects on visual prognosis, with sPED at baseline being more associated with PED resolution after anti-VEGF therapy.^61^ Our study showed a significant positive association between sPED and VA and a significant negative association between fvPED and VA in first- and second-treated eyes. As discussed, the association of fvPED and poorer VA could be explained by a later presentation of a more advanced neovascular AMD process. Serous PED is more common in younger age groups who present at an earlier stage of the disease process, and this might correspond to better VA. Drusen being more common in second-treated eyes also directly correlated with a better VA. Although not included in current re-treatment protocols, sub-RPE activity seems to precede degenerative cystic formation, and its recurrence has been linked to the primary event of neovascular reactivation and long-term vision loss.^62^ It has been suggested that the increase in PED volume during early stages of anti-VEGF therapy is a useful indicator of fluid recurrence,^63^ and the presence of PED may be predictive of more regular treatment.^60^

Central subfield thickness had the highest association with VA in first-treated eyes. At baseline, higher CST usually correlates with poor VA, but this correlation becomes less evident during follow-up.^64^^,^^65^ Therefore, although used in re-treatment decisions of major clinical trials, use of CST has been questioned because of poor reproducibility and lack of correlation with visual outcomes after treatment.^34^ A well-known limitation is that the CST sums several different retinal structures, each structure independently affecting functional outcomes. One could argue that if IRF, SRF, and SHRM all have some degree of negative correlation with VA, when analyzing them together in the form of CST, a stronger association can be observed compared with analyzing each of them individually. However, this once again highlights the importance of segmenting different features within the total OCT volume scan.

### Study Limitations

The limitations for this study include its retrospective nature, the variability in the time that patients present, and the lack of reading center grading for the individual segmented features. Additionally, we have not included the location of the segmented features within the retinal volume, which could provide further insights into the pathophysiology of the disease and visual prognosis.^66^ In future reports, we intend to analyze this further and provide retinal layer information including axial location and distance to the foveal center. Furthermore, stratifying our cohort to analyze ethnic differences in neovascular AMD generated unequal group sizes because of the greater prevalence of AMD in White populations.^67^^,^^68^ Although these results reflect outcomes from a diverse set of patients from Moorfields Eye Hospital, it does not fully represent a global population. Considering the epidemiology of AMD as a multifactorial disease for which genetics, race, diet, and lifestyle play a role in disease development, additional studies using diverse datasets would be ideal to compare analyses.

The segmentation outputs from this study have been made openly available for the ophthalmic and AMD research community to download together with this article. This endorses the worldwide effort to inspire community progress in the healthcare sector. We compared our results with prior work that calculated tissue and fluid volumes and thicknesses (Table S2, available at www.aaojournal.org). Discrepancies observed could arise from differences in methodologies, study design, and data interpretation, for example, the use of different OCT devices and scan protocols, as well as the difference in cohort demographics. Therefore, making this comprehensive dataset openly available will be particularly interesting for ophthalmologists to compare our findings on the baseline OCT characteristics of a large real-world cohort with those from clinical trials. This could help the clinical community determine whether these trials have enrolled patients who are representative of real-world practice. Additionally, it will also allow others to replicate our findings and conduct their own novel analyses. Potential clinical uses of the segmentation system may include diagnosis and stratification of neovascular AMD. In uncertain cases and recent conversion to neovascular AMD, the system could detect and quantify subtle or high-risk features of exudation. In addition, quantification of volumes may aid monitoring efficacy of treatment, provide insight to aid anti-VEGF drug choice, and help optimize re-treatment intervals. Furthermore, the system could allow clinicians to see where the eye in question is in terms of the usual spectrum of eyes with neovascular AMD seen in real-world practice.

In this study, we presented the results of a large-scale analysis using an automated deep learning 3D segmentation system that classifies and quantifies multiple features within an OCT volume scan. Our large cohort was extracted from the Moorfields AMD database, which is perhaps the largest single-center dataset of patients with neovascular AMD.^28^ Automating OCT segmentation will become crucial in further understanding disease subgroups and quantifying disease progression at a patient level. The characterization and quantification of several features may aid personalized medicine and suggest novel anatomic parameters that can unravel new structure–function correlations in neovascular AMD.

## References

[1] Lai T.-T., Hsieh Y.-T., Yang C.-M. (2019). Biomarkers of optical coherence tomography in evaluating the treatment outcomes of neovascular age-related macular degeneration: a real-world study. Sci Rep.

[2] Schmidt-Erfurth U., Waldstein S.M. (2016). A paradigm shift in imaging biomarkers in neovascular age-related macular degeneration. Prog Retin Eye Res.

[3] Waldstein S.M., Philip A.-M., Leitner R. (2016). Correlation of 3-dimensionally quantified intraretinal and subretinal fluid with visual acuity in neovascular age-related macular degeneration. JAMA Ophthalmol.

[4] Fung A.E., Lalwani G.A., Rosenfeld P.J. (2007). An optical coherence tomography-guided, variable dosing regimen with intravitreal ranibizumab (Lucentis) for neovascular age-related macular degeneration. Am J Ophthalmol.

[5] Lalwani G.A., Rosenfeld P.J., Fung A.E. (2009). A variable-dosing regimen with intravitreal ranibizumab for neovascular age-related macular degeneration: year 2 of the PrONTO Study. Am J Ophthalmol.

[6] Holz F.G., Amoaku W., Donate J. (2011). Safety and efficacy of a flexible dosing regimen of ranibizumab in neovascular age-related macular degeneration: the SUSTAIN study. Ophthalmology.

[7] Boyer D.S., Heier J.S., Brown D.M. (2009). A Phase IIIb study to evaluate the safety of ranibizumab in subjects with neovascular age-related macular degeneration. Ophthalmology.

[8] Sadda S.R., Wu Z., Walsh A.C. (2006). Errors in retinal thickness measurements obtained by optical coherence tomography. Ophthalmology.

[9] Waldstein S.M., Gerendas B.S., Montuoro A. (2015). Quantitative comparison of macular segmentation performance using identical retinal regions across multiple spectral-domain optical coherence tomography instruments. Br J Ophthalmol.

[10] Simader C., Ritter M., Bolz M. (2014). Morphologic parameters relevant for visual outcome during anti-angiogenic therapy of neovascular age-related macular degeneration. Ophthalmology.

[11] Waldstein S.M., Simader C., Staurenghi G. (2016). Morphology and visual acuity in aflibercept and ranibizumab therapy for neovascular age-related macular degeneration in the VIEW Trials. Ophthalmology.

[12] Waldstein S.M., Wright J., Warburton J. (2016). Predictive value of retinal morphology for visual acuity outcomes of different ranibizumab treatment regimens for neovascular AMD. Ophthalmology.

[13] Willoughby A.S., Ying G.-S., Toth C.A. (2015). Subretinal hyperreflective material in the d. Ophthalmology.

[14] Busbee B.G., Ho A.C., Brown D.M. (2013). Twelve-month efficacy and safety of 0.5 mg or 2.0 mg ranibizumab in patients with subfoveal neovascular age-related macular degeneration. Ophthalmology.

[15] Toth C.A., Decroos F.C., Ying G.-S. (2015). Identification of fluid on optical coherence tomography by treating ophthalmologists versus a reading center in the comparison of age-related macular degeneration treatments trials. Retina.

[16] Zheng Y., Sahni J., Campa C. (2013). Computerized assessment of intraretinal and subretinal fluid regions in spectral-domain optical coherence tomography images of the retina. Am J Ophthalmol.

[17] Schmidt-Erfurth U., Vogl W.-D., Jampol L.M., Bogunović H. (2020). Application of automated quantification of fluid volumes to anti-VEGF therapy of neovascular age-related macular degeneration. Ophthalmology.

[18] Lee H., Kang K.E., Chung H., Kim H.C. (2018). Automated segmentation of lesions including subretinal hyperreflective material in neovascular age-related macular degeneration. Am J Ophthalmol.

[19] Li M.-X., Yu S.-Q., Zhang W. (2019). Segmentation of retinal fluid based on deep learning: application of three-dimensional fully convolutional neural networks in optical coherence tomography images. Int J Ophthalmol.

[20] Gao K., Niu S., Ji Z. (2019). Double-branched and area-constraint fully convolutional networks for automated serous retinal detachment segmentation in SD-OCT images. Comput Methods Programs Biomed.

[21] Lu D., Heisler M., Lee S. (2019). Deep-learning based multiclass retinal fluid segmentation and detection in optical coherence tomography images using a fully convolutional neural network. Med Image Anal.

[22] Lee C.S., Tyring A.J., Deruyter N.P. (2017). Deep-learning based, automated segmentation of macular edema in optical coherence tomography. Biomed Opt Express.

[23] Bogunovic H., Venhuizen F., Klimscha S. (2019). RETOUCH: the retinal OCT fluid detection and segmentation benchmark and challenge. IEEE Trans Med Imaging.

[24] Schlegl T., Waldstein S.M., Bogunovic H. (2018). Fully automated detection and quantification of macular fluid in OCT using deep learning. Ophthalmology.

[25] Guymer R.H., Markey C.M., McAllister I.L. (2019). Tolerating subretinal fluid in neovascular age-related macular degeneration treated with ranibizumab using a treat-and-extend regimen: FLUID study 24-month results. Ophthalmology.

[26] De Fauw J., Ledsam J.R., Romera-Paredes B. (2018). Clinically applicable deep learning for diagnosis and referral in retinal disease. Nat Med.

[27] Yim J., Chopra R., Spitz T. (2020). Predicting conversion to wet age-related macular degeneration using deep learning. Nat Med.

[28] Fasler K., Moraes G., Wagner S. (2019). One- and two-year visual outcomes from the Moorfields age-related macular degeneration database: a retrospective cohort study and an open science resource. BMJ Open.

[29] Fasler K., Fu D.J., Moraes G. (2020). Moorfields AMD database report 2: fellow eye involvement with neovascular age-related macular degeneration. Br J Ophthalmol.

[30] Huang J., Liu X., Wu Z. (2009). Macular thickness measurements in normal eyes with time-domain and Fourier-domain optical coherence tomography. Retina.

[31] Lee H., Jo A., Kim H.C. (2017). Three-dimensional analysis of morphologic changes and visual outcomes in neovascular age-related macular degeneration. Invest Ophthalmol Vis Sci.

[32] Ristau T., Keane P.A., Walsh A.C. (2014). Relationship between visual acuity and spectral domain optical coherence tomography retinal parameters in neovascular age-related macular degeneration. Ophthalmologica.

[33] Keane P.A., Chang K.T., Liakopoulos S. (2009). Effect of ranibizumab retreatment frequency on neurosensory retinal volume in neovascular AMD. Retina.

[34] Keane P.A., Liakopoulos S., Chang K.T. (2008). Relationship between optical coherence tomography retinal parameters and visual acuity in neovascular age-related macular degeneration. Ophthalmology.

[35] Joeres S., Tsong J.W., Updike P.G. (2007). Reproducibility of quantitative optical coherence tomography subanalysis in neovascular age-related macular degeneration. Invest Ophthalmol Vis Sci.

[36] Hu X., Waldstein S.M., Klimscha S. (2020). Morphological and functional characteristics at the onset of exudative conversion in age-related macular degeneration. Retina.

[37] Heier J.S., Brown D.M., Chong V. (2012). Intravitreal aflibercept (VEGF trap-eye) in wet age-related macular degeneration. Ophthalmology.

[38] Martin D.F., Maguire M.G., CATT Research Group (2011). Ranibizumab and bevacizumab for neovascular age-related macular degeneration. N Engl J Med.

[39] Dugel P.U., Koh A., Ogura Y. (2020). HAWK and HARRIER: phase 3, multicenter, randomized, double-masked trials of brolucizumab for neovascular age-related macular degeneration. Ophthalmology.

[40] Pontikos N., Wagner S.K., Balaskas K. (2020). Correspondence: Trends in retina specialist imaging utilization from 2012 to 2016 in the United States Medicare fee-for-service population. Am J Ophthalmol.

[41] Schmidt-Erfurth U., Sadeghipour A., Gerendas B.S. (2018). Artificial intelligence in retina. Prog Retin Eye Res.

[42] Torkamani A., Andersen K.G., Steinhubl S.R., Topol E.J. (2017). High-definition medicine. Cell.

[43] Spaide R.F., Jaffe G.J., Sarraf D. (2020). Consensus nomenclature for reporting neovascular age-related macular degeneration data: Consensus on Neovascular Age-Related Macular Degeneration Nomenclature Study Group. Ophthalmology.

[44] Owen C.G., Jarrar Z., Wormald R. (2012). The estimated prevalence and incidence of late stage age related macular degeneration in the UK. Br J Ophthalmol.

[45] Sharma H.E., Mathewson P.A., Lane M. (2014). The role of social deprivation in severe neovascular age-related macular degeneration. Br J Ophthalmol.

[46] Holz F.G., Tadayoni R., Beatty S. (2016). Key drivers of visual acuity gains in neovascular age-related macular degeneration in real life: findings from the AURA study. Br J Ophthalmol.

[47] Freund K.B., Zweifel S.A., Engelbert M. (2010). Do we need a new classification for choroidal neovascularization in age-related macular degeneration?. Retina.

[48] Cheung C.M.G., Lai T.Y.Y., Ruamviboonsuk P. (2018). Polypoidal choroidal vasculopathy: definition, pathogenesis, diagnosis, and management. Ophthalmology.

[49] Jaffe G.J., Martin D.F., Toth C.A. (2013). Macular morphology and visual acuity in the comparison of age-related macular degeneration treatments trials. Ophthalmology.

[50] Ritter M., Simader C., Bolz M. (2014). Intraretinal cysts are the most relevant prognostic biomarker in neovascular age-related macular degeneration independent of the therapeutic strategy. Br J Ophthalmol.

[51] Sharma S., Toth C.A., Daniel E. (2016). Macular morphology and visual acuity in the second year of the Comparison of Age-Related Macular Degeneration Treatments Trials. Ophthalmology.

[52] Grunwald J.E., Daniel E., Huang J. (2014). Risk of geographic atrophy in the comparison of age-related macular degeneration treatments trials. Ophthalmology.

[53] Chevreaud O., Semoun O., Blanco-Garavito R. (2016). Visual acuity at presentation in the second eye versus first eye in patients with exudative age-related macular degeneration. Eur J Ophthalmol.

[54] Peyman M., Peyman A., Lansingh V.C. (2019). Intravitreal bevacizumab versus ranibizumab: effects on the vessels of the fellow non-treated eye. J Curr Ophthalmol.

[55] Avery R.L., Castellarin A.A., Steinle N.C. (2017). Systemic pharmacokinetics and pharmacodynamics of intravitreal aflibercept, bevacizumab, and ranibizumab. Retina.

[56] Shah V.P., Shah S.A., Mrejen S., Freund K.B. (2014). Subretinal hyperreflective exudation associated with neovascular age-related macular degeneration. Retina.

[57] Segal O., Barayev E., Nemet A.Y. (2016). Prognostic value of hyperreflective foci in neovascular age-related macular degeneration treated with bevacizumab. Retina.

[58] Akagi-Kurashige Y., Tsujikawa A., Oishi A. (2012). Relationship between retinal morphological findings and visual function in age-related macular degeneration. Graefes Arch Clin Exp Ophthalmol.

[59] Lee H., Ji B., Chung H., Kim H.C. (2016). Correlation between optical coherence tomographic hyperreflective foci and visual outcomes after anti-VEGF treatment in neovascular age-related macular degeneration and polypoidal choroidal vasculopathy. Retina.

[60] Cheong K.X., Chong Teo K.Y., Ming Cheung G.C. (2020 May 16). Influence of pigment epithelial detachment on visual acuity in neovascular age-related macular degeneration. Surv Ophthalmol.

[61] Cho H.J., Kim K.M., Kim H.S. (2016). Response of pigment epithelial detachment to anti-vascular endothelial growth factor treatment in age-related macular degeneration. Am J Ophthalmol.

[62] Schmidt-Erfurth U., Waldstein S.M., Deak G.-G. (2015). Pigment epithelial detachment followed by retinal cystoid degeneration leads to vision loss in treatment of neovascular age-related macular degeneration. Ophthalmology.

[63] Penha F.M., Gregori G., Garcia Filho CA. de A. (2013). Quantitative changes in retinal pigment epithelial detachments as a predictor for retreatment with anti-VEGF therapy. Retina.

[64] Zhang X., Lai T.Y.Y. (2018). Baseline predictors of visual acuity outcome in patients with wet age-related macular degeneration. Biomed Res Int.

[65] Ou W.C., Brown D.M., Payne J.F., Wykoff C.C. (2017). Relationship between visual acuity and retinal thickness during anti-vascular endothelial growth factor therapy for retinal diseases. Am J Ophthalmol.

[66] Wu Z., Cunefare D., Chiu E. (2016). Longitudinal associations between microstructural changes and microperimetry in the early stages of age-related macular degeneration. Invest Ophthalmol Vis Sci.

[67] Mohamed R., Gadhvi K., Mensah E. (2018). What effect does ethnicity have on the response to ranibizumab in the treatment of wet age-related macular degeneration?. Ophthalmologica.

[68] Patel S.N., Wu C., Obeid A. (2020). Sociodemographic factors in neovascular age-related macular degeneration. Ophthalmology.

