# Supplementary material for: Quantitative Analysis of OCT for Neovascular Age-Related Macular Degeneration Using Deep Learning
Source: Ophthalmology. 2021 May;128(5):693–705. doi: 10.1016/j.ophtha.2020.09.025 (PMC8528155; doi:10.1016/j.ophtha.2020.09.025)
Supplement: Fig S1 [file mmc1.pdf]

Confusion matrices for agreement between experts on fluid presence

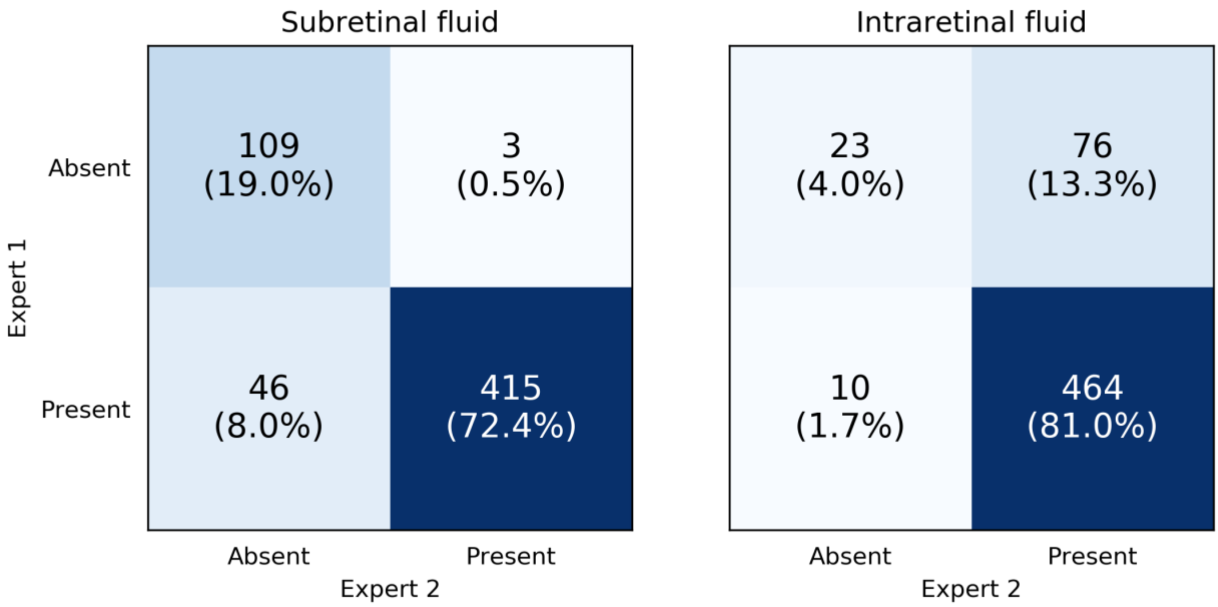

sFigure 1. Confusion matrices showing agreement between experts for subretinal fluid (SRF) and intraretinal fluid (IRF). Of 573 scans, the experts agreed on the presence or absence in 91.4% and 85.0% of scans for SRF and IRF, respectively.
