# Supplementary material for: Quantitative Analysis of OCT for Neovascular Age-Related Macular Degeneration Using Deep Learning
Source: Ophthalmology. 2021 May;128(5):693–705. doi: 10.1016/j.ophtha.2020.09.025 (PMC8528155; doi:10.1016/j.ophtha.2020.09.025)
Supplement: Fig S2 [file mmc2.pdf]

ROC curves for presence of IRF/SRF

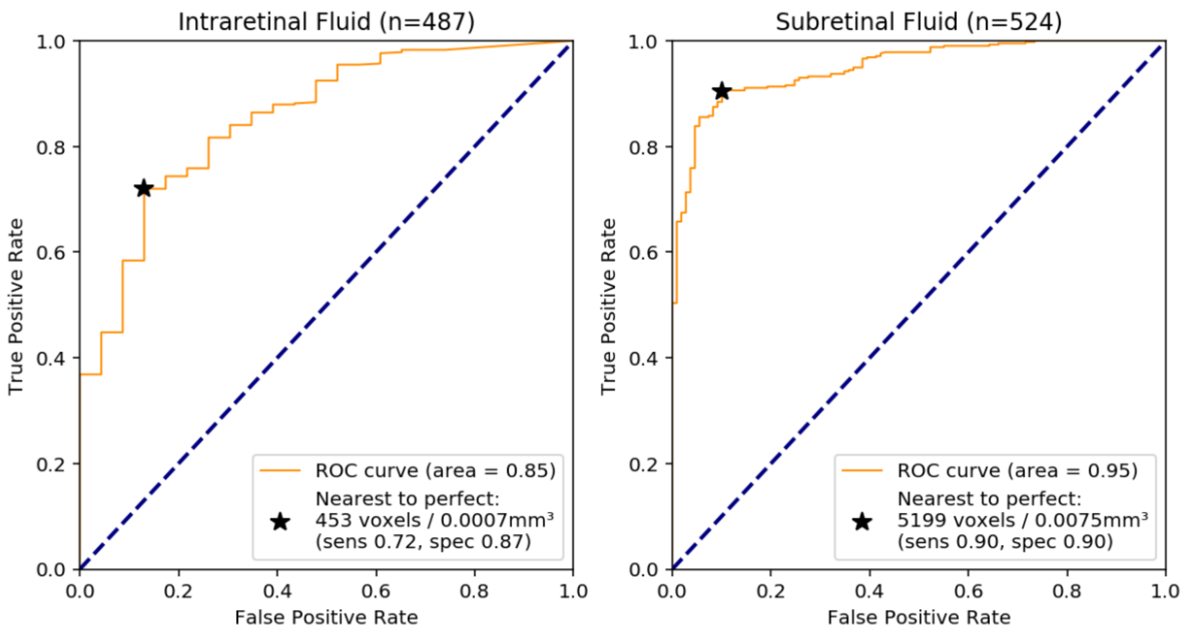

sFigure 2. Receiver operating characteristic curves (ROC) for left: intraretinal fluid, and right: subretinal fluid, plotting the diagnostic accuracy of the segmentation model, using only scans where the retinal specialists unanimously agreed. The operating point closest to the top left corner was chosen to define fluid presence. This corresponds to a threshold of ≥453 voxels (0.0007mm_3) and ≥5199 (0.0075mm_3) voxels for IRF and SRF respectively. IRF = intraretinal fluid, SRF = subretinal fluid, ROC = Receiver operating characteristic.
