# Supplementary material for: Quantitative Analysis of OCT for Neovascular Age-Related Macular Degeneration Using Deep Learning
Source: Ophthalmology. 2021 May;128(5):693–705. doi: 10.1016/j.ophtha.2020.09.025 (PMC8528155; doi:10.1016/j.ophtha.2020.09.025)
Supplement: Fig S3 [file mmc3.pdf]

Confusion matrices for agreement between ground truth and the model classification at the chosen operating point for fluid presence

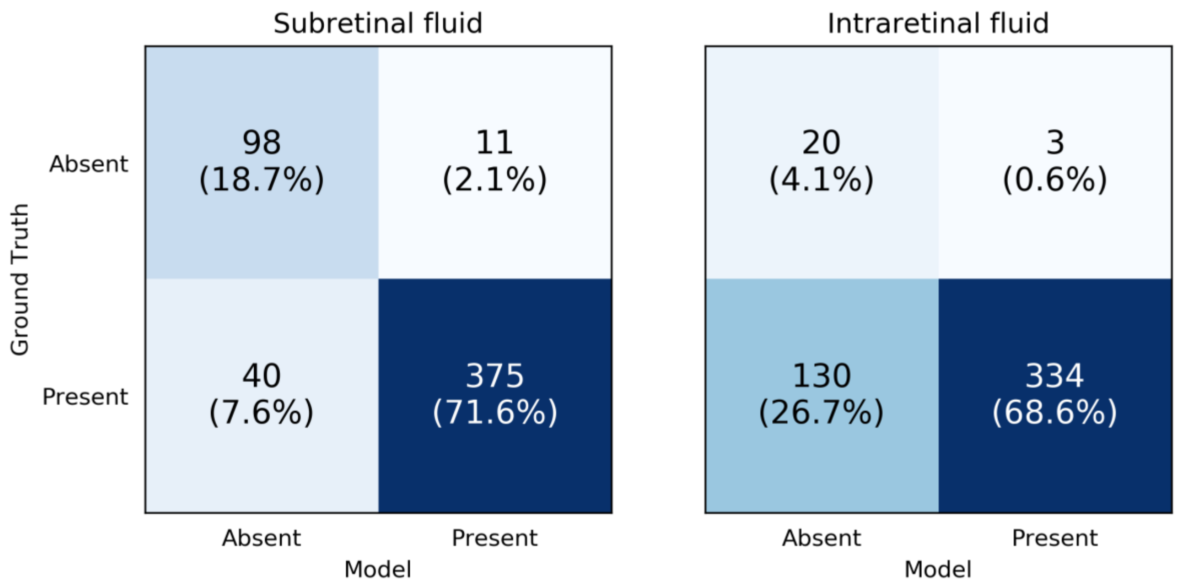

sFigure3 - Confusion matrices showing agreement between the ground truth (where the two experts agreed) and the model classification at the chosen operating point for subretinal fluid (SRF) and intraretinal fluid (IRF). Of 524 scans where the experts agreed on presence or absence of SRF, the model also agreed in 90.3% of scans. Of 487 scans where the experts agreed on presence or absence of IRF, the model also agreed in 72.7% of scans.
