# Supplementary material for: Quantitative Analysis of OCT for Neovascular Age-Related Macular Degeneration Using Deep Learning
Source: Ophthalmology. 2021 May;128(5):693–705. doi: 10.1016/j.ophtha.2020.09.025 (PMC8528155; doi:10.1016/j.ophtha.2020.09.025)
Supplement: Fig S4 [file mmc4.pdf]

Scatter plot comparing central subfield thickness with visual acuity in first-treated eyes

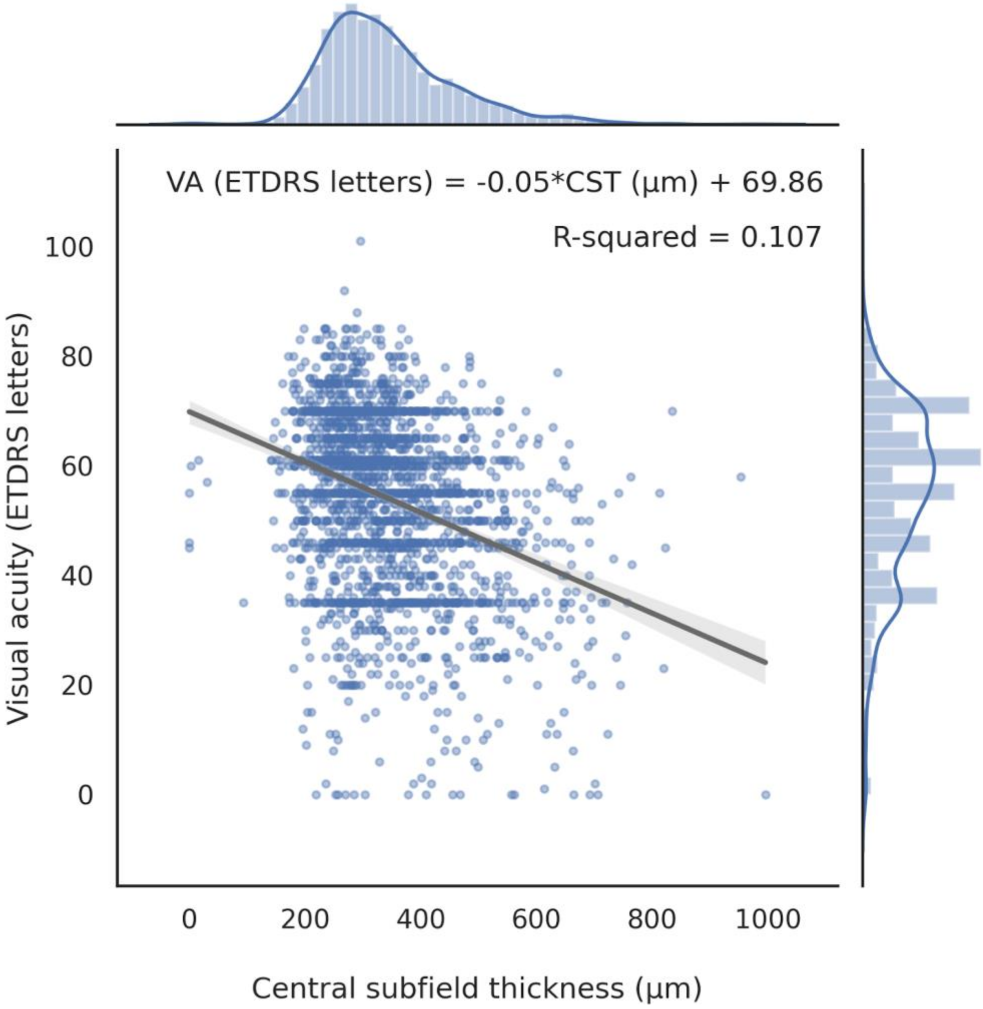

sFigure 4. Scatter plot comparing CST with visual acuity in first-treated eyes. The regression line: VA (ETDRS letters) = -0.04 * CST (μm) + 69.78, and 95% confidence intervals (shaded) are shown. ETDRS = Early treatment diabetic retinopathy study, VA = Visual acuity, CST = central subfield thickness.
