# Supplementary material for: Quantitative Analysis of OCT for Neovascular Age-Related Macular Degeneration Using Deep Learning
Source: Ophthalmology. 2021 May;128(5):693–705. doi: 10.1016/j.ophtha.2020.09.025 (PMC8528155; doi:10.1016/j.ophtha.2020.09.025)
Supplement: Fig S5 [file mmc5.pdf]

Scatter plot comparing subretinal hyperreflective material with visual acuity in second-treated eyes

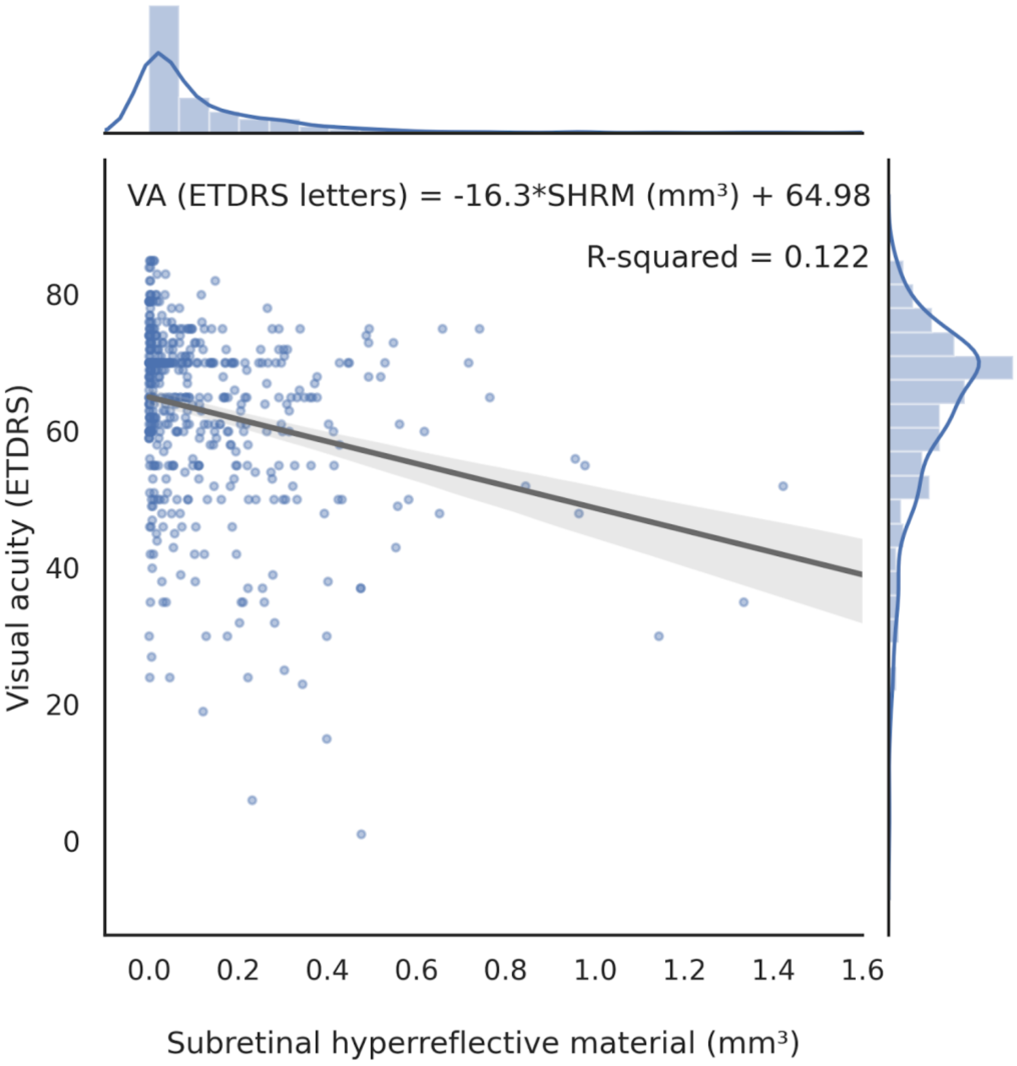

sFigure 5. Scatter plot comparing subretinal hyperreflective material with visual acuity in second-treated eyes. The regression line: VA (ETDRS letters) = -16.3 * SHRM (mm$_3$) + 64.98, and 95% confidence intervals (shaded) are shown. ETDRS = Early treatment diabetic retinopathy study, SHRM = Subretinal hyperreflective material, VA = Visual acuity.
