# Supplementary material for: Quantitative Analysis of OCT for Neovascular Age-Related Macular Degeneration Using Deep Learning
Source: Ophthalmology. 2021 May;128(5):693–705. doi: 10.1016/j.ophtha.2020.09.025 (PMC8528155; doi:10.1016/j.ophtha.2020.09.025)
Supplement: Table S1 [file mmc6.pdf]

Mean or median volumes of OCT segmented features at baseline in patients with both first and second-treated eyes in the dataset

Segmented feature	Mean (standard deviation) at first injection		Median (interquartile range) at first injection		Wilcoxon signed rank test P-value
	First-treated eye	Second- treated eye	First-treated eye	Second-treated eye	
**NSR volume (mm$_3$)**	**9.507 (0.945)**	9.270 (0.780)	**9.500 (8.941–10.022)**	9.315 (8.784–9.774)	0.160
**RPE volume (mm$_3$)**	**0.800 (0.079)**	0.792 (0.089)	**0.803 (0.760–0.852)**	0.801 (0.755–0.845)	**<0.001**
**IRF volume (mm$_3$)**	**0.142 (0.328)**	0.072 (0.201)	**0.009 (0.000–0.115)**	0.004 (0.000–0.044)	**<0.001**
**SRF volume (mm$_3$)**	**0.457 (0.725)**	0.235 (0.478)	**0.191 (0.023–0.624)**	0.046 (0.005–0.242)	**<0.001**
**SHRM volume (mm$_3$)**	**0.375 (0.574)**	0.142 (0.290)	**0.159 (0.036–0.462)**	0.054 (0.006–0.174)	**0.003**
**HRF volume (mm$_3$)**	**0.003 (0.008)**	0.002 (0.003)	0.001 (0.000–0.003)	**0.001 (0.000–0.002)**	**<0.001**
**Drusen volume (mm$_3$)**	0.035 (0.065)	**0.064 (0.085)**	0.013 (0.004–0.041)	**0.033 (0.010–0.088)**	**<0.001**
**fvPED volume (mm$_3$)**	**0.921 (1.274)**	0.485 (0.977)	**0.414 (0.135–1.104)**	0.194 (0.062–0.479)	**0.002**
**sPED volume (mm$_3$)**	**0.003 (0.013)**	0.002 (0.012)	**0.000 (0.000–0.001)**	0.000 (0.000–0.000)	**<0.001**
**CST (µm)**	**353.7 (110.2)**	306.3 (80.2)	**341.0 (243.5–409.9)**	297.1 (256.9–340.3)	**<0.001**

sTable 1. Mean and median volumes with standard deviations and interquartile range of segmented features in individuals with both first- and second-treated eyes in the dataset (n=387). Volumes given at first injection. Segmented voxels were converted into mm$_3$. P-values were considered significant at ≤0.05. NSR = neurosensory retina, RPE = retinal pigment epithelium, IRF = intraretinal fluid, SRF = subretinal fluid, PED = pigment epithelium detachment, SHRM = subretinal hyperreflective material, HRF = hyperreflective foci, fvPED = fibrovascular PED, sPED = serous PED, CST = central subfield thickness.
