# Supplementary material for: Quantitative Analysis of OCT for Neovascular Age-Related Macular Degeneration Using Deep Learning
Source: Ophthalmology. 2021 May;128(5):693–705. doi: 10.1016/j.ophtha.2020.09.025 (PMC8528155; doi:10.1016/j.ophtha.2020.09.025)
Supplement: Table S3 [file mmc7.pdf]

Comparison of mean central subfield thickness at baseline among different published studies

Study	Moorfields AMD first-treated eye	VIEW 1 [65]	VIEW 2 [65]	PRONTO [4]	CATT[66]
CST definition	Average thickness in the central circle of 1mm diameter, as defined by ETDRS	Not reported	Not reported	Average thickness in the central circle of 1mm diameter, as defined by ETDRS	Single point measurement using callipers at the fovea
OCT Device	Spectral-domain 3DOCT-2000 (Topcon Corp., Tokyo, Japan)	Time-domain Stratus (Carl Zeiss Meditec, Jena, Germany)	Time-domain Stratus (Carl Zeiss Meditec, Jena, Germany)	Time-domain Stratus (Carl Zeiss Meditec, Dublin, California, USA)	Time-domain OCT
CST (µm)	347.1	313.2 - 325.9*	326.5 - 342.6*	393.9	247 - 252*
**Study**	**SUSTAIN [6]**	**SAILOR [7]**	**HARBOR [14]**	**HAWK [67]**	**HARRIER[67]**
CST definition	Not reported	Not reported	Not reported	Average thickness in the central circle of 1mm diameter, as defined by ETDRS	Average thickness in the central circle of 1mm diameter, as defined by ETDRS
OCT Device	Not reported	Not reported	Spectral-domain Cirrus HD-OCT III (Carl Zeiss Meditec, Inc., Dublin, CA)	Spectral-domain OCT	Spectral-domain OCT
CST (µm)	340.5	312 - 322*	332 - 348*	457.9 - 466.6*	465.3 - 473.6*

sTable 3. Central subfield thickness (CST) measurements at baseline. The first treated eye is used for analysis. This is compared with other clinical trials that have reported CST in a similar cohort. CST = Central subfield thickness, OCT = optical coherence tomography. *CST mean baseline measurements vary in this range depending on the treatment arm.
