# Supplementary material for: Quantitative Analysis of OCT for Neovascular Age-Related Macular Degeneration Using Deep Learning
Source: Ophthalmology. 2021 May;128(5):693–705. doi: 10.1016/j.ophtha.2020.09.025 (PMC8528155; doi:10.1016/j.ophtha.2020.09.025)
Supplement: Table S4 [file mmc8.docx]

**Supplementary Table 4 (sTable 4)**

**First-treated eye mean volumes by different subgroups**

|  | **Mean volume, mm^3^ (standard deviation)** | | | |
| --- | --- | --- | --- | --- |
| **Visual acuity (ETDRS letters)** | **0-35** | **36-52** | **53-69** | **≥70** |
| **NSR** | 9.738 (1.421) | 9.508 (1.014) | 9.383 (0.890) | 9.426 (0.759) |
| **RPE** | 0.786 (0.092) | 0.803 (0.084) | 0.806 (0.087) | 0.829 (0.083) |
| **IRF** | 0.228 (0.459) | 0.164 (0.343) | 0.082 (0.222) | 0.050 (0.249) |
| **SRF** | 0.588 (0.912) | 0.470 (0.721) | 0.436 (0.723) | 0.383 (0.653) |
| **SHRM** | 0.709 (0.856) | 0.463 (0.733) | 0.291 (0.551) | 0.173 (0.420) |
| **HRF** | 0.005 (0.011) | 0.003 (0.008) | 0.003 (0.008) | 0.002 (0.006) |
| **Drusen** | 0.024 (0.049) | 0.032 (0.097) | 0.039 (0.094) | 0.046 (0.085) |
| **fvPED** | 1.123 (1.738) | 0.896 (1.422) | 0.675 (1.147) | 0.551 (1.047) |
| **sPED** | 0.002 (0.013) | 0.003 (0.012) | 0.004 (0.023) | 0.008 (0.036) |
| **CST *** | 403.3 (137.9) | 370.9 (120.6) | 328.7 (102.0) | 301.8 (77.8) |
|  |  |  |  |  |
|  | **Mean volume, mm^3^ (standard deviation)** | | | |
| **Age (years)** | **50-59** | **60-69** | **70-79** | **≥80** |
| **NSR** | 9.653 (1.116) | 9.624 (0.721) | 9.538 (1.000) | 9.415 (1.064) |
| **RPE** | 0.856 (0.072) | 0.838 (0.084) | 0.818 (0.079) | 0.789 (0.090) |
| **IRF** | 0.093 (0.234) | 0.080 (0.235) | 0.107 (0.360) | 0.134 (0.291) |
| **SRF** | 0.744 (1.037) | 0.471 (0.699) | 0.487 (0.727) | 0.419 (0.724) |
| **SHRM** | 0.401 (0.485) | 0.330 (0.615) | 0.368 (0.599) | 0.396 (0.710) |
| **HRF** | 0.003 (0.005) | 0.003 (0.008) | 0.003 (0.008) | 0.003 (0.009) |
| **Drusen** | 0.026 (0.056) | 0.038 (0.127) | 0.035 (0.081) | 0.037 (0.077) |
| **fvPED** | 0.719 (1.179) | 0.755 (1.326) | 0.870 (1.566) | 0.707 (1.120) |
| **sPED** | 0.019 (0.066) | 0.007 (0.021) | 0.005 (0.029) | 0.002 (0.011) |
| **CST *** | 372.0 (151.2) | 341.3 (106.3) | 346.5 (113.0) | 347.5 (114.8) |
|  |  |  |  |  |
|  | **Mean volume, mm^3^ (standard deviation)** | | | |
| **Race/Ethnicity** | **White** | **Asian** | **Black** | **Other or unknown** |
| **NSR** | 9.481 (0.982) | 9.436 (1.112) | 9.363 (0.922) | 9.514 (1.035) |
| **RPE** | 0.803 (0.090) | 0.807 (0.078) | 0.847 (0.077) | 0.807 (0.088) |
| **IRF** | 0.112 (0.272) | 0.150 (0.423) | 0.127 (0.287) | 0.118 (0.322) |
| **SRF** | 0.436 (0.706) | 0.433 (0.755) | 0.754 (0.924) | 0.471 (0.749) |
| **SHRM** | 0.375 (0.678) | 0.356 (0.533) | 0.442 (0.620) | 0.390 (0.667) |
| **HRF** | 0.003 (0.008) | 0.003 (0.008) | 0.004 (0.008) | 0.003 (0.009) |
| **Drusen** | 0.038 (0.098) | 0.044 (0.086) | 0.023 (0.037) | 0.032 (0.063) |
| **fvPED** | 0.718 (1.169) | 0.857 (1.762) | 0.690 (1.125) | 0.815 (1.353) |
| **sPED** | 0.003 (0.013) | 0.005 (0.023) | 0.014 (0.066) | 0.005 (0.028) |
| **CST *** | 345.7 (110.4) | 343.4 (122.5) | 360.7 (122.6) | 349.6 (117.2) |

**sTable 4**. Mean first-treated eye volumes for segmented features stratified by age, visual acuity and race/ethnicity subgroups. *CST measures thickness and not volume. IRF = intraretinal fluid, SRF = subretinal fluid, SHRM = subretinal hyperreflective material, NSR = neurosensory retina, HRF = Hyperreflective foci, RPE = retinal pigment epithelium, sPED = serous pigment epithelium detachment, fvPED = fibrovascular pigment epithelium detachment, ETDRS = Early treatment diabetic retinopathy study, CST = Central subfield thickness.
