# Supplementary material for: Quantitative Analysis of OCT for Neovascular Age-Related Macular Degeneration Using Deep Learning
Source: Ophthalmology. 2021 May;128(5):693–705. doi: 10.1016/j.ophtha.2020.09.025 (PMC8528155; doi:10.1016/j.ophtha.2020.09.025)
Supplement: Table S4 [file mmc9.pdf]

First-treated eye mean volumes by different subgroups

	Mean volume, mm^3 (standard deviation)			
**Visual acuity (ETDRS letters)**	**0-35**	**36-52**	**53-69**	**≥70**
**NSR**	9.738 (1.421)	9.508 (1.014)	9.383 (0.890)	9.426 (0.759)
**RPE**	0.786 (0.092)	0.803 (0.084)	0.806 (0.087)	0.829 (0.083)
**IRF**	0.228 (0.459)	0.164 (0.343)	0.082 (0.222)	0.050 (0.249)
**SRF**	0.588 (0.912)	0.470 (0.721)	0.436 (0.723)	0.383 (0.653)
**SHRM**	0.709 (0.856)	0.463 (0.733)	0.291 (0.551)	0.173 (0.420)
**HRF**	0.005 (0.011)	0.003 (0.008)	0.003 (0.008)	0.002 (0.006)
**Drusen**	0.024 (0.049)	0.032 (0.097)	0.039 (0.094)	0.046 (0.085)
**fvPED**	1.123 (1.738)	0.896 (1.422)	0.675 (1.147)	0.551 (1.047)
**sPED**	0.002 (0.013)	0.003 (0.012)	0.004 (0.023)	0.008 (0.036)
**CST ***	403.3 (137.9)	370.9 (120.6)	328.7 (102.0)	301.8 (77.8)

	Mean volume, mm^3 (standard deviation)			
**Age (years)**	**50-59**	**60-69**	**70-79**	**≥80**
**NSR**	9.653 (1.116)	9.624 (0.721)	9.538 (1.000)	9.415 (1.064)
**RPE**	0.856 (0.072)	0.838 (0.084)	0.818 (0.079)	0.789 (0.090)
**IRF**	0.093 (0.234)	0.080 (0.235)	0.107 (0.360)	0.134 (0.291)
**SRF**	0.744 (1.037)	0.471 (0.699)	0.487 (0.727)	0.419 (0.724)
**SHRM**	0.401 (0.485)	0.330 (0.615)	0.368 (0.599)	0.396 (0.710)
**HRF**	0.003 (0.005)	0.003 (0.008)	0.003 (0.008)	0.003 (0.009)
**Drusen**	0.026 (0.056)	0.038 (0.127)	0.035 (0.081)	0.037 (0.077)
**fvPED**	0.719 (1.179)	0.755 (1.326)	0.870 (1.566)	0.707 (1.120)
**sPED**	0.019 (0.066)	0.007 (0.021)	0.005 (0.029)	0.002 (0.011)
**CST ***	372.0 (151.2)	341.3 (106.3)	346.5 (113.0)	347.5 (114.8)

	Mean volume, mm^3 (standard deviation)			
**Race/Ethnicity**	**White**	**Asian**	**Black**	**Other or unknown**
**NSR**	9.481 (0.982)	9.436 (1.112)	9.363 (0.922)	9.514 (1.035)

**RPE**	0.803 (0.090)	0.807 (0.078)	0.847 (0.077)	0.807 (0.088)
**IRF**	0.112 (0.272)	0.150 (0.423)	0.127 (0.287)	0.118 (0.322)
**SRF**	0.436 (0.706)	0.433 (0.755)	0.754 (0.924)	0.471 (0.749)
**SHRM**	0.375 (0.678)	0.356 (0.533)	0.442 (0.620)	0.390 (0.667)
**HRF**	0.003 (0.008)	0.003 (0.008)	0.004 (0.008)	0.003 (0.009)
**Drusen**	0.038 (0.098)	0.044 (0.086)	0.023 (0.037)	0.032 (0.063)
**fvPED**	0.718 (1.169)	0.857 (1.762)	0.690 (1.125)	0.815 (1.353)
**sPED**	0.003 (0.013)	0.005 (0.023)	0.014 (0.066)	0.005 (0.028)
**CST ***	345.7 (110.4)	343.4 (122.5)	360.7 (122.6)	349.6 (117.2)

sTable 4. Mean first-treated eye volumes for segmented features stratified by age, visual acuity and race/ethnicity subgroups. *CST measures thickness and not volume. IRF = intraretinal fluid, SRF = subretinal fluid, SHRM = subretinal hyperreflective material, NSR = neurosensory retina, HRF = Hyperreflective foci, RPE = retinal pigment epithelium, sPED = serous pigment epithelium detachment, fvPED = fibrovascular pigment epithelium detachment, ETDRS = Early treatment diabetic retinopathy study, CST = Central subfield thickness.
