# Supplementary material for: Quantitative Analysis of OCT for Neovascular Age-Related Macular Degeneration Using Deep Learning
Source: Ophthalmology. 2021 May;128(5):693–705. doi: 10.1016/j.ophtha.2020.09.025 (PMC8528155; doi:10.1016/j.ophtha.2020.09.025)
Supplement: Table S5 [file mmc10.pdf]

Multivariable linear regression analysis for visual acuity in first-treated eyes

	Model A Coefficient (standard error)	Model B Coefficient (standard error)	Model C Coefficient (standard error)	Model D Coefficient (standard error)
**Constant**	28.356** (2.902)	28.371** (2.900)	27.837** (2.692)	27.868** (2.693)
**NSR**	-0.204 (0.416)	-0.206 (0.416)		
**RPE**	37.843** (4.317)	37.894** (4.307)	36.902** (3.812)	37.451** (3.795)
**IRF**	-4.808** (1.173)	-4.807** (1.173)	-4.996** (1.109)	-4.970** (1.109)
**SRF**	0.952* (0.474)	0.945* (0.472)	0.955* (0.471)	1.003* (0.470)
**SHRM**	-3.194** (0.570)	-3.198** (0.570)	-3.215** (0.568)	-3.275** (0.567)
**HRF**	-73.729 (37.588)	-73.734 (37.580)	-74.292* (37.557)	-75.009* (37.563)
**Drusen**	0.644 (3.604)			
**fvPED**	-1.714** (0.264)	-1.714** (0.264)	-1.708** (0.263)	-1.689** (0.263)
**sPED**	19.420 (13.249)	19.370 (13.243)	19.494 (13.239)	
**CST**	-0.036** (0.004)	-0.036** (0.004)	-0.037** (0.004)	-0.037** (0.004)
**Age**	-0.127* (0.037)	-0.127** (0.037)	-0.126* (0.037)	-0.130** (0.037)
**Female**	14.117** (1.490)	14.127** (1.488)	13.859** (1.386)	13.880** (1.386)
**Male**	14.239** (1.480)	14.244** (1.480)	13.978** (1.379)	13.988** (1.379)
**Asian**	6.095** (1.106)	6.105** (1.104)	5.959** (1.064)	5.931** (1.064)
**Black**	5.833* (1.746)	5.830* (1.745)	5.770* (1.741)	5.873* (1.740)
**Other or unknown**	6.965** (0.981)	6.967** (0.981)	6.804** (0.924)	6.800** (0.924)
**White**	9.464** (0.991)	9.469** (0.990)	9.305** (0.932)	9.264** (0.932)
**R-squared**	0.214	0.214	0.214	0.213
**Adjusted R-squared**	**0.209**	**0.209**	**0.209**	**0.209**
**Number of observations**	2247			

sTable 5. Multivariable linear regression analysis for visual acuity in ETDRS letters in first-treated eyes. Using a process of backwards elimination, each model removed a single variable with the highest P-value until all remaining variables were significant ($P \le 0.05$). All variables were included in model A. The variable with the highest P-value, drusen was removed for model B. Model C additionally excluded neurosensory retina (NSR), and model D additionally eliminated serous PED (sPED). The final model (D) includes 14 variables with $P \le 0.05$ with an adjusted R-squared of 0.209. *$P \le 0.05$, **$P \le 0.001$. IRF = intraretinal fluid, SRF = subretinal fluid, SHRM = subretinal hyperreflective material, NSR = neurosensory retina, HRF = Hyperreflective foci, RPE = retinal pigment epithelium, sPED = serous pigment epithelium detachment, fvPED = fibrovascular pigment epithelium detachment, ETDRS = Early treatment diabetic retinopathy study, CST = Central subfield thickness.
