# Supplementary material for: Quantitative Analysis of OCT for Neovascular Age-Related Macular Degeneration Using Deep Learning
Source: Ophthalmology. 2021 May;128(5):693–705. doi: 10.1016/j.ophtha.2020.09.025 (PMC8528155; doi:10.1016/j.ophtha.2020.09.025)
Supplement: Table S6 [file mmc11.pdf]

Comparison of binary fluid presence at baseline among different studies

Parameter	Moorfields AMD first-treated eye	Moorfields AMD second-treated eye	VIEW 1/2[11]	PRONTO[4]	CATT[43]	HAWK[67]	HARRIER[67]
**IRF (%)**	66.8	60.2	69.0-72.3*	90	70.1-83.2*	53.9-54.7*	37.7-40.3*
**SRF (%)**	82.7	72.6	82.9-87.6*	75	79.2-87.3*	68.1-69.4*	67.8-72.6*

sTable 6. Percentage of eyes classified with each feature compared to other clinical trials that used manual grading methodologies. For our study, a threshold was used to determine the presence or absence of each feature while in clinical trials this was done through manual grading. IRF and SRF are defined as present at ≥36 voxels and ≥2018 voxels, respectively. AMD = age related macular degeneration, IRF = intraretinal fluid, SRF = subretinal fluid. * percentages vary among treatment arms.
