# Supplementary material for: Quantitative Analysis of OCT for Neovascular Age-Related Macular Degeneration Using Deep Learning
Source: Ophthalmology. 2021 May;128(5):693–705. doi: 10.1016/j.ophtha.2020.09.025 (PMC8528155; doi:10.1016/j.ophtha.2020.09.025)
Supplement: Table S2 [file mmc12.pdf]

Mean or median volumes of OCT segmented features at baseline among different published studies

<table>
<tr><th colspan="2"></th><th>Moorfields AMD first-treated eye</th><th>Waldstein et al [3]</th><th>Lee et al [40]</th><th>Keane et al [53]</th><th>Joeres et al [63]</th><th>Keane et al [54]</th><th>Hu et al [64]</th><th>Ristau et al [45]</th></tr>
<tr><td colspan="2">Scan area (mm)</td><td>6 x 6</td><td>6 x 6</td><td>9 x 6</td><td>6 mm (radial lines scan protocol)</td><td>6 mm (radial lines scan protocol)</td><td>6 mm (radial lines scan protocol)</td><td>6 x 6</td><td>15 x 20</td></tr>
<tr><td colspan="2">Region for volume calculations</td><td>Entire scan area</td><td>Entire scan area</td><td>Within 500 µm radius of fovea</td><td>Entire scan area</td><td>Entire scan area</td><td>Entire scan area</td><td>Entire scan area</td><td>Within 500 µm radius of fovea</td></tr>
<tr><td colspan="2">Number of OCT B-scans</td><td>128</td><td>128</td><td>25</td><td>6</td><td>6</td><td>6</td><td>128</td><td>37</td></tr>
<tr><td colspan="2">Statistical measure</td><td>Mean (SD)</td><td>Mean (SD)</td><td>Mean (SD)</td><td>Mean (SD)</td><td>Mean</td><td>Mean (SD)</td><td>Median</td><td>Mean</td></tr>
<tr><td rowspan="6">Baseline segmented features (volumes, mm^3)</td><td>IRF</td><td>0.118 (0.31)</td><td>0.105 (0.19)</td><td>0.03 (0.05)</td><td></td><td></td><td></td><td>0.021</td><td></td></tr>
<tr><td>SRF</td><td>0.455 (0.73)</td><td>0.341 (0.45)</td><td>0.04 (0.06)</td><td>0.36 (0.60)</td><td>0.19</td><td>0.46 (0.65)</td><td>0.012</td><td>0.04</td></tr>
<tr><td>fvPED</td><td>0.765 (1.31)</td><td></td><td>0.04 (0.05)</td><td rowspan="2">1.01 (1.76)</td><td rowspan="2">1.04</td><td rowspan="2">0.70 (1.26)</td><td rowspan="2">0.094</td><td rowspan="2">0.07</td></tr>
<tr><td>sPED</td><td>0.004 (0.02)</td><td></td><td>0.01 (0.04)</td></tr>
<tr><td>Drusen</td><td>0.036 (0.09)</td><td></td><td></td><td></td><td></td><td></td><td></td><td></td></tr>
<tr><td>SHRM</td><td>0.380 (0.67)</td><td></td><td>0.04 (0.06)</td><td>0.30 (0.52)</td><td>0.32</td><td>0.33 (0.53)</td><td>0.000</td><td>0.03</td></tr>
</table>

sTable 2. Mean or median segmented feature volumes at baseline. In our study, the first treated eye is used for the analysis. This is compared to other studies that present some volumetric calculations for a neovascular AMD cohort prior to the start of treatment. AMD = Age related macular degeneration, OCT = optical coherence tomography, SD = standard deviation, IRF = intraretinal fluid, SRF = subretinal fluid, fvPED = fibrovascular pigment epithelium detachment, sPED = serous pigment epithelium detachment, SHRM = subretinal hyperreflective material.
